# Prevalence and molecular characterization of cefotaxime-resistant *Salmonella* strains recovered from retail meat samples in Shenzhen, China, during 2014–2017

**DOI:** 10.1128/spectrum.04886-22

**Published:** 2023-08-24

**Authors:** Chen Yang, Kaichao Chen, Lianwei Ye, Heng Heng, Xuemei Yang, Edward Wai-chi Chan, Sheng Chen

**Affiliations:** 1 Department of Infectious Diseases and Public Health, Jockey Club College of Veterinary Medicine and Life Sciences, City University of Hong Kong, Kowloon, Hong Kong; 2 State Key Lab of Chemical Biology and Drug Discovery and the Department of Food Science and Nutrition, The Hong Kong Polytechnic University, Hung Hom, China; 3 Shenzhen Key Lab for Biological Safety Control, The Hong Kong Polytechnic University Shenzhen Research Institute, Shenzhen, China; Yangzhou University, Yangzhou, China

**Keywords:** *Salmonella*, CTX-M, cefotaxime, multidrug resistance, plasmid, food

## Abstract

In this work, we collected foodborne *Salmonella* strains in Shenzhen, China, during 2014–2017 and investigated the genetic profile of all cefotaxime-resistant isolates in the collection. The strains were subjected to antimicrobial susceptibility tests, whole-genome sequencing, bioinformatics analysis, and conjugation studies. A total of 79 cefotaxime-resistant *Salmonella* were identified and found to exhibit multidrug resistance. Resistance rate recorded during the study period increased from 1.9% to 9.1%. *Salmonella* Typhimurium was the predominant serovar, and CTX-M family genes were dominant among the ESBLs genes detected. Notably, CTX-M-bearing plasmids or transposons often contain other drug resistance genes. Furthermore, a combination of CTX-M-55 and CTX-M-65 genes was detected for the first time in foodborne *Salmonella* strains. Our findings reveal the prevalence and molecular characteristics of cefotaxime-resistant foodborne *Salmonella* strains in southern China.

**IMPORTANCE:**

Cefotaxime-resistant *Salmonella* strains pose an increasing threat to human health by causing infections with limited treatment options. It is therefore necessary to undertake a surveillance on the prevalence of such strains and investigate the resistance and transmission mechanisms. In this work, various ESBL genes flanked by different IS located in different mobile genetic elements were detectable among cefotaxime-resistant *Salmonella* strains. These data show that the high prevalence and genotypic diversity of cefotaxime-resistant foodborne *Salmonella* strains in China are possibly attributed to the evolution and transmission of a wide range of multidrug resistance-encoding mobile genetic elements.

## INTRODUCTION

Cefotaxime was discovered in 1976 and marketed as a “third-generation” cephalosporin in 1980. It was synthesized by adding an α-syn-methoxy-imino group to an aminothiazoyl ring that contains an acetyl side chain (1). Like other cephalosporins, cefotaxime binds to penicillin-binding proteins Ib and III through the β-lactam ring, inhibiting the process of transpeptidation in bacterial cell wall synthesis of bacteria and causing autolysis of bacteria (1). Cefotaxime exhibits a broad spectrum of antimicrobial activity against anaerobic, Gram-positive, and Gram-negative bacteria, especially strains that produce β-lactamase to degrade caphalosporins of the previous generations. Besides, cefotaxime does not cause coagulopathies and pseudocholelithiasis (2). Therefore, this drug is extremely effective in the treatment of serious infections caused by Gram-negative bacterial pathogens.

*Salmonella*, which belongs to the *Enterobacteriaceae* family, is a leading cause of foodborne illnesses worldwide and a reservoir of antibiotic resistance genes due to its ability to inhabit a wide range of animal hosts, including pigs, cattle, poultry, wildlife, and companion animals (3 – 5). The predominant source of community-acquired *Salmonella* infection is food animals, from which *Salmonella* may come into contact with human through the activities of animal husbandry, food delivery, processing, retail, home handling, and ingestion of contaminated food. *Salmonella* infections are usually self-limited, but may develop into systemic infections of high mortality rate in a small proportion of patients, such as the immunocompromised and elderly patients. In such cases, antibiotics are required for treatment. Cefotaxime is an important antibiotic commonly used to treat *Salmonella* infections. However, incidence of cefotaxime resistance has increased significantly in the past decade, presumably due to the selection pressure created as a result of increased use of cefotaxime in the treatment of *Salmonella* infection in both human and food animals. The molecular mechanisms of cefotaxime resistance in *Salmonella* have been under intensive investigation (4, 6). The major resistance mechanism involves hydrolysis of cefotaxime by the production of extended-spectrum beta lactamases (ESBLs). The most prevalent ESBLs produced by *Salmonella* are CTX-M, TEM, and SHV. Among the enzymes of the CTX-M family, CTX-M-15 is dominant worldwide, while CTX-M-14 is the most prevalent in China. CTX-M-27 was found to exhibit a striking increase in prevalence globally, including China, since it was identified in 2003 (6). The AmpC β-lactamase-encoding gene *bla*_CMY-2_ was the most important ESBL gene before the CTX-M-encoding gene became the most common ESBL gene worldwide except for China (7, 8).

In this work, we conducted a surveillance on the prevalence of cefotaxime resistance among *Salmonella* strains isolated from retail pork, chicken, beef, and shrimp during 2014–2017 and performed whole-genome sequencing to investigate the molecular mechanisms of cefotaxime resistance among such strains.

## MATERIALS AND METHODS

### Isolation of *Salmonella* from retail meat products

Retail samples of pork, chicken, beef, and shrimp were purchased from supermarkets and wet markets in Shenzhen, China, during 2014–2017. *Salmonella* strains were isolated as described previously (9). Typical *Salmonella* strains recovered from each sample were purified and subjected to species identification by the detection of *invA* gene and by using the matrix-assisted laser desorption ionization-time of flight mass spectrometry biotyper system (Bruker, Germany). All isolates were serotyped according to the Kauffmann–White scheme using commercial antiserum (Difco, Detroit, MI) (10).

### Antimicrobial susceptibility tests

*Salmonella* isolates were subjected to the assessment of susceptibility to 12 antimicrobial drugs using the agar dilution and broth microdilution method based on the 29th CLSI and EUCAST (v 12.0) guidelines (11, 12). *Staphylococcus aureus* ATCC 29213 and *Escherichia coli* ATCC 25922 were used as quality control strains. The 12 antimicrobial compounds tested were ampicillin, cefotaxime, ceftriaxone, meropenem, ciproﬂoxacin, nalidixic acid, azithromycin, chloramphenicol, sulfamethoxazole-trimethoprim, tetracycline, amikacin, and kanamycin. Minimal inhibitory concentration (MIC) of the test drugs was determined in at least three experiments for each strain.

### Genomic sequencing and analysis

*Salmonella* genomic DNA was extracted from each test isolate using the PureLink Genomic DNA Mini Kit (Invitrogen, United States) according to the manufacturer’s instructions. DNA libraries were constructed by using the NEBNext Ultra II DNA Library Preparation Kit for Illumina (New England Biolabs, United States) and sequenced via the 150 bp paired-end NextSeq 500 platform (Illumina, San Diego, CA). Raw reads were trimmed and quality filtered using Trimmomatic v0.36 (13). Draft genomes of the test strains were assembled using SPAdes v3.10.1 (14). To identify the genetic features of antibiotic resistance genes and assess the distribution patterns of such genes in the plasmids, draft genome searches were conducted by using ResFinder (15), PlasmidFinder (16), and the CLC Genomics Workbench (CLC bio, Denmark). To compare the plasmids identified in this work with structurally similar plasmids carrying cefotaxime resistance genes that were reported in previous studies, all structurally similar plasmid sequences were downloaded from the National Center for Biotechnology Information (NCBI) database upon BLASTn screening with contigs containing cefotaxime resistance gene against the nonredundant protein sequence (nr) database. Genomic DNA from strain SA535 was subjected to sequencing with the long-read MinION platform, following the manufacturer’s guideline (Oxford Nanopore Technologies, Oxford, United Kingdom). Both long and short reads were *de novo* hybrid assembled using Unicycler v0.4.8 (17). Assembled genome sequences were annotated by RAST (18), ResFinder (15), ISFinder (19), PlasmidFinder (16), and the CLC Genomics Workbench (CLC bio, Denmark). Alignment of plasmid sequences with similar structures was performed by BLAST Ring Image Generator (BRIG) v0.95.22 (20) and Easyfig_win_2.1 (21). Whole-genome phylogenetic trees containing reference isolates were created for the identification of 14-Sa44. Single-nucleotide polymorphisms (SNPs) were generated using Snippy v3.1 with default settings (22), and whole-genome alignment was used to infer the MLST phylogenies using Fasttree v2.1.10, with default parameters (23). The phylogenetic tree was visualized by iTOL version 5 (24).

### Conjugation and plasmid characterization

The transmission potential of the cefotaxime resistance genes was assessed by performing the conjugation experiment using the filter mating method as previously described, with slight modifications (25, 26). *Salmonella* strains that harbored the cefotaxime resistance genes were used as donor strain, and sodium azide-resistant *E. coli J53* was used as the recipient strain. Transconjugants were selected on eosin methylene blue agar containing sodium azide (100 µg/mL) and cefotaxime (2 µg/mL). The presence of the cefotaxime resistance genes as a marker gene in the plasmid harbored by the transconjugants was determined by PCR (26). The MIC profiles of the transconjugants were determined to confirm that they differed from those of the donor. The sizes of large plasmids of *Salmonella* isolates and corresponding transconjugants were determined by S1-PFGE as previously described (25).

## RESULTS

### Prevalence of cefotaxime resistant *Salmonella* strains in retail meat products

A total of 1,038 non-duplicate *Salmonella* strains were isolated from 2,975 food samples (1,711 pork, 529 chicken, 294 beef, and 441 shrimp samples) collected in Shenzhen, China, during 2014–2017. Among the *Salmonella* strains isolated from pork, beef, and chicken samples, 79 exhibited cefotaxime resistance (MIC ≥4); the resistance rate was 1.9% (3/157) in 2014 and 2.4% (7/286) in 2015, but increased to 12.4% (56/452) in 2016 before dropping to 9.1% (13/143) in 2017 (Table S1); 74.68% of the cefotaxime-resistant *Salmonella* strains were isolated from pork, followed by chicken (21.52%), beef (2.53%), and shrimp (1.27%) (Table 1). The 79 cefotaxime-resistant *Salmonella* strains were found to belong to 13 serotypes, namely, the predominant *Salmonella enterica* serovar Typhimurium (*S*. Typhimurium) (59.49%) and its monophasic variant *S*. 4,(5),12:i:- (2.53%), *S*. Albany (10.13%), *S*. Indiana (8.86%), *S*. Rissen (3.80%), *S*. Kentucky (2.53%), *S*. London (2.53%), *S*. Stanley (2.53%), *S*. Weltevreden (2.53%), *S*. Meleagridis (1.27%), *S*. Derby (1.27%), *S*. Parkroyal (1.27%), and *S*. Senftenberg (1.27%) (Table 2).

**TABLE 1:** Genetic and phenotypic characteristics of 79 cefotaxime-resistant foodborne *Salmonella* strains^*a*^

				MIC				Target mutation				
ID	Year of isolation	Serotypes	Source of isolation	CTX	CIP	AZI	β-Lactamase	*gyrA*	*parC*	Other important acquired resistance genes	Plasmid types	Estimated size
14-Sa44	2014	London	Pork	>16	2	>32	CTX-M-130	−	−	qnrB6, qnrS1, mph(A)	IncFIB/l1	202,750
14-Sa54	2014	Indiana	Pork	>16	>16	>32	CTX-M-65	+	+	oqxAB, erm(T)	IncHI2	228,062
14-Sa115	2014	Albany	Pork	>16	2	2	CTX-M-130	+	−		Incl1	91,411
SA535	2015	Rissen	Pork	>16	0.03	<0.25	CTX-M-27	+	+		IncP	62620
SA560	2015	Kentucky	Chicken	>16	16	1	CTX-M-14	+	+	qnrS1		
SA567	2015	Indiana	Chicken	>16	>16	32	CTX-M-14	+	+	qnrS1, oqxAB, mph(A)	IncN	85,943
SA583	2015	Typhimurium	Pork	16	0.12	<0.25	CTX-M-14	−	−	qnrS1, oqxAB, mcr-1.1	IncHI2	247,705
SA617	2015	Typhimurium	Pork	>16	0.25	0.5	CTX-M-14	−	−	qnrS1, oqxAB, mcr-1.1	IncHI2	247,705
SA627	2015	Typhimurium	Pork	>16	4	1	CTX-M-14	+	−	qnrS1, oqxAB	IncHI2	247,705
SA727	2015	Typhimurium	Pork	>16	1	1	CTX-M-55	−	−	qnrB19, qnrS1	Transposon	8,993
SA745	2016	Weltevreden	Pork	>16	1	1	CTX-M-65	−	−	qnrS1	IncHI2	228,062
SA748	2016	Weltevreden	Pork	>16	1	1	CTX-M-65	−	−	qnrS1	IncHI2	228,062
SA795	2016	Indiana	Chicken	>16	>16	>32	CTX-M-14	+	+	qnrS1, oqxAB, mph(A)	IncN	85,943
SA796	2016	Indiana	Chicken	>16	>16	>32	CTX-M-14	+	+	qnrS1, oqxAB, mph(A)	IncN	85,943
SA797	2016	Indiana	Chicken	>16	>16	>32	CTX-M-14	+	+	qnrS1, oqxAB, mph(A)	IncN	85,943
SA837	2016	Albany	Chicken	>16	1	2	CTX-M-55	+	−	qnrB19, qnrS1		
SA896	2016	Parkroyal	Pork	>16	>16	32	CTX-M-55	+	−	qnrS1, oqxAB, mph(A)	IncHI2	257,945
SA902	2016	Typhimurium	Pork	>16	0.5	1	CTX-M-55	−	−	qnrS1	Transposon	8,993
SA910	2016	Rissen	Pork	>16	0.03	1	CTX-M-65	+	+		IncFII	186,499
SA927	2016	Senftenberg	Pork	>16	1	1	CTX-M-14	−	−	qnrS1	IncHI1	244,597
SA938	2016	Typhimurium	Pork	>16	0.12	4	CTX-M-14	−	−	qnrS1, mcr-1.1	IncHI2	247,705
SA945	2016	Typhimurium	Pork	>16	0.5	1	CTX-M-55	−	−	qnrS1	Transposon	8,993
SA956	2016	Typhimurium	Pork	>16	4	2	CTX-M-14	+	−	qnrS1, oqxAB, mcr-1.1	IncHI2	247,705
SA1034	2016	Typhimurium	Pork	>16	1	1	CTX-M-55	−	−	qnrS1	Transposon	8,993
SA1048	2016	Typhimurium	Pork	>16	2	1	CTX-M-14	+	−	qnrS1, oqxAB, mcr-1.1	IncHI2	247,705
SA1069	2016	Typhimurium	Pork	16	0.03	1	CTX-M-14	−	−	oqxAB, mcr-1.1	IncHI2	247,705
SA1077	2016	Albany	Chicken	>16	1	2	CTX-M-55	−	−			
SA1085	2016	Typhimurium	Pork	>16	0.06	1	CTX-M-14	−	−	oqxAB, mcr-1.1	IncHI2	247,705
SA1092	2016	Typhimurium	Pork	16	0.06	1	CTX-M-14	−	−	qnrS1, oqxAB, mcr-1.1	IncHI2	247,705
SA1105	2016	Meleagridis	Chicken	>16	1	16	CTX-M-55	−	−	qnrB6, qnrS1, mph(A)		
SA1158	2016	Kentucky	Chicken	>16	16	0.5	CTX-M-14	+	+	qnrS1		
SA1226	2016	Typhimurium	Pork	>16	2	2	CTX-M-14	+	−	qnrS1, oqxAB, mcr-1.1	IncHI2	247,705
SA1258	2016	Typhimurium	Pork	>16	0.12	0.5	CTX-M-14	−	−	oqxAB, mcr-1.1	IncHI2	247,705
SA1265	2016	Albany	Pork	>16	1	1	CTX-M-55	+	−	qnrS1		
SA1266	2016	Typhimurium	Pork	>16	0.5	0.5	CTX-M-55	−	−	qnrS1	Transposon	8,993
SA1276	2016	Typhimurium	Pork	>16	0.5	1	CTX-M-55	−	−	qnrS1	Transposon	8,993
SA1285	2016	Typhimurium	Pork	>16	0.5	1	CTX-M-55	−	−	qnrS1	Transposon	8,993
SA1286	2016	Albany	Chicken	>16	1	1	CTX-M-55	+	−	qnrS11		
SA1287	2016	Albany	Chicken	>16	1	1	CTX-M-55	+	−	qnrS1		
SA1302	2016	Typhimurium	Pork	>16	0.5	1	CTX-M-55	−	−	qnrS1	Transposon	8,993
SA1330	2016	Typhimurium	Pork	>16	0.5	1	CTX-M-55	−	−	qnrS1	Transposon	8,993
SA1334	2016	Typhimurium	Pork	>16	0.5	1	CTX-M-55	−	−	qnrS1	Transposon	8,993
SA1352	2016	Typhimurium	Pork	>16	0.5	1	CTX-M-55	−	−	qnrS1	Transposon	8,993
SA1362	2016	Albany	Pork	>16	0.5	2	CTX-M-55	+	−			
SA1363	2016	Albany	Chicken	>16	1	2	CTX-M-55	+	−			
SA1416	2016	Stanley	Pork	>16	2	16	CTX-M-55	−	−	qnrS1, mph(A)	IncHI2	257,945
SA1441	2016	Stanley	Shrimp	>16	0.5	0.25	CTX-M-55	−	−	qnrS1		
SA1465	2016	4,[5],12:i:-	Pork	>16	1	1	CTX-M-14	+	−	oqxAB, mcr-1.1	IncHI2	247,705
SA1512	2016	Typhimurium	Pork	>16	>16	8	CTX-M-14	+	−	oqxAB, mcr-1.1	IncHI2	247,705
SA1522	2016	Typhimurium	Beef	>16	1	1	CTX-M-65	−	−	qnrS1	IncHI2	228,062
SA1523	2016	Typhimurium	Pork	>16	1	0.5	CTX-M-65	−	−	qnrS1	IncHI2	228,062
SA1527	2016	Typhimurium	Pork	>16	2	2	CTX-M-14	+	−	oqxAB, mcr-1.1	IncHI2	247,705
SA1532	2016	Typhimurium	Beef	>16	1	2	CTX-M-65	−	−	qnrS1	IncHI2	228,062
SA1536	2016	Typhimurium	Pork	>16	4	2	CTX-M-14	+	−	qnrS1, qnrVC5, oqxAB, mcr-1.1	IncHI2	247,705
SA1541	2016	Typhimurium	Pork	>16	0.5	0.5	CTX-M-55/65	−	−	qnrS1	IncHI2	228,062
SA1547	2016	Typhimurium	Pork	>16	1	1	CTX-M-65	−	−	qnrS1, qnrVC5	IncHI2	228,062
SA1551	2016	Typhimurium	Chicken	>16	1	0.5	CTX-M-65	−	−	qnrS1	IncHI2	228,062
SA1559	2016	Typhimurium	Pork	>16	1	1	CTX-M-65	−	−	qnrS1	IncHI2	228,062
SA1561	2016	Typhimurium	Pork	>16	1	0.5	CTX-M-65	−	−	qnrS1	IncHI2	228,062
SA1562	2016	Typhimurium	Pork	>16	1	1	CTX-M-65	−	−	qnrS1	IncHI2	228,062
SA1567	2016	Derby	Pork	>16	8	0.5	CMY-2	−	−	qnrS2, oqxAB	IncFII	98,807
SA1574	2016	Typhimurium	Pork	>16	1	1	CTX-M-65	−	−	qnrS1	IncHI2	228,062
SA1596	2016	Typhimurium	Pork	>16	0.5	0.5	CTX-M-55	−	−	qnrS1	Transposon	8,993
SA1629	2016	Typhimurium	Pork	>16	0.5	1	CTX-M-55	−	−	qnrS1	Transposon	8,993
SA1649	2016	Indiana	Chicken	>16	16	0.5	CTX-M-14	+	+	qnrS13, oqxAB	IncHI2	247,705
SA1719	2016	Typhimurium	Pork	8	1	>32	CTX-M-14	+	−	oqxAB, mph(A), mcr-1.1	IncHI2	247,705
SA1848	2017	4,[5],12:i:-	Pork	>16	1	0.5	CTX-M-65	−	−	qnrS1	IncHI2	228,062
SA1850	2017	Typhimurium	Pork	>16	2	8	CTX-M-14	−	−	qnrS1	IncHI2	247,705
SA1852	2017	Typhimurium	Pork	>16	8	0.5	CTX-M-65	−	−	qnrS1, qnrS2, oqxAB	IncHI2	228,062
SA1853	2017	London	Pork	>16	1	0.5	CTX-M-65	−	−	qnrB6		
SA1857	2017	Typhimurium	Pork	>16	0.5	1	CTX-M-55	−	−	qnrS1	Transposon	8,993
SA1969	2017	Typhimurium	Chicken	>16	>16	16	CTX-M-14	+	−	oqxAB	IncHI2	247,705
SA1973	2017	Typhimurium	Pork	>16	>16	8	CTX-M-65	+	−	oqxAB	IncHI2	228,062
SA1974	2017	Typhimurium	Pork	>16	1	1	CTX-M-65	−	−	qnrS1	IncHI2	228,062
SA1976	2017	Indiana	Chicken	>16	1	1	CTX-M-55	+	+	qnrS1	IncHI2	257,945
SA2004	2017	Rissen	Chicken	>16	>16	1	CMY-2	+	−		IncFII	98,807
SA2041	2017	Typhimurium	Pork	>16	4	1	CTX-M-14	+	−	oqxAB, mcr-1.1	IncHI2	247,705
SA2074	2017	Typhimurium	Pork	>16	0.5	2	CTX-M-55	−	−	qnrS1	Transposon	8,993
SA2075	2017	Typhimurium	Pork	>16	1	1	CTX-M-55	−	−	qnrS1	Transposon	8,993

^*a*^ CTX, Cefotaxime; CIP, Ciprofloxacin; AZI, Azithromycin.

**TABLE 2:** Rate of cefotaxime resistance among strains of various *Salmonella* serovars^*a*^

	No. of CTX^r^ isolates/No. of isolates (%)				
Serovar	2014	2015	2016	2017	Total
Typhimurium	0/22 (0.0)	4/38 (10.5)	34/89 (38.2)	9/32 (28.1)	47/181 (26.0)
Albany	1/3 (33.3)	0/1 (0.0)	7/9 (77.8)	0/0 (0.0)	8/13 (61.5)
Indiana	1/4 (25.0)	1/5 (20.0)	4/7 (57.1)	1/4 (25.0)	7/20 (35.0)
Rissen	0/20 (0.0)	1/41 (2.4)	1/24 (4.2)	1/14 (7.1)	3/99 (3.0)
Kentucky	0/0 (0.0)	1/6 (16.7)	1/12 (5.9)	0/15 (0.0)	2/38 (5.3)
London	1/20 (5.0)	0/10 (0.0)	0/22 (0.0)	1/9 (11.1)	2/61 (3.3)
4,[5],12:i:-	0/0 (0.0)	0/0 (0.0)	1/2 (50.0)	1/4 (25.0)	2/6 (33.3)
Stanley	0/5 (0.0)	0/12 (0.0)	2/7 (28.6)	0/1 (0.0)	2/25 (8.0)
Weltevreden	0/3 (0.0)	0/9 (0.0)	2/3 (66.7)	0/1 (0.0)	2/16 (12.5)
Meleagridis	0/9 (0.0)	0/2 (0.0)	1/7 (14.3)	0/3 (0.0)	1/21 (4.8)
Derby	0/52 (0.0)	0/74 (0.0)	1/81 (1.2)	0/30 (0.0)	1/237 (0.4)
Parkroyal	0/0 (0.0)	0/0 (0.0)	1/1 (100.0)	0/0 (0.0)	1/1 (100.0)
Senftenberg	0/0 (0.0)	0/5 (0.0)	1/9 (11.0)	0/0 (0.0)	1/14 (7.1)

^*a*^ CTXr: Cefotaxime resistant.

**Fig 1:**
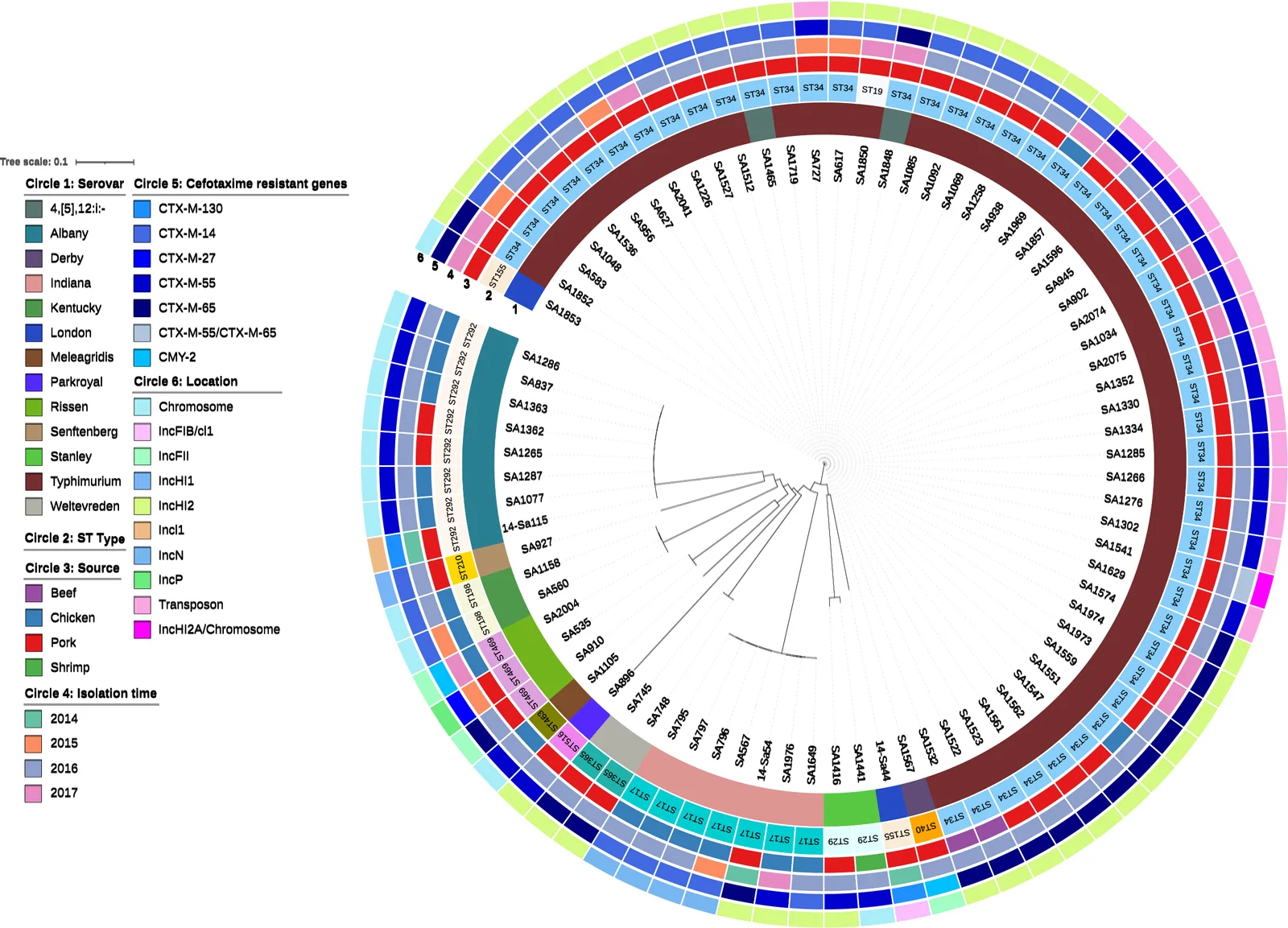
The phylogenetic tree of 79 cefotaxime-resistant foodborne *Salmonella* strains recovered during 2014–2017. From the inside to the outside, each circle represents the serovar, ST types, source, isolation time, cefotaxime-resistant genes, and distribution of cefotaxime-resistant genes, respectively.

Among the 181 *S*. Typhimurium strains, none of the isolates recovered from 2014 was resistant to cefotaxime, yet the resistance rate was found to increase from 10.5% in 2015 to 38.3% in 2016, but decreased to 25.0% in 2017. The cefotaxime resistance rate of the other two prevalent serotypes, *S*. Rissen and *S*. Derby, was 3.0% (3/99) and 0.4% (1/237), respectively, which is much lower than that of *S*. Typhimurium (Table 2). *S*. Typhimurium accounted for 70% (*n* = 42) of pork-borne cefotaxime-resistant *Salmonella* strains and was the only serovar detectable in beef-borne cefotaxime-resistant *Salmonella* isolates. Among the 17 cefotaxime-resistant *Salmonella* strains isolated from chicken samples, the two most common serotypes were *S*. Indiana (29.4%) and *S*. Albany (29.4%). The unique shrimp-borne cefotaxime-resistant *Salmonella* strain belonged to the serotype *S*. Stanley. The MLST results showed that most of the *S*. Typhimurium and its monophasic variant isolates belonged to the same sequence type, namely, ST34, and that only one *S*. Typhimurium strain was ST19. Among strains of the other serovars, the relationship between the serovar and the MLST profile of each strain was found to be unique and tightly linked, with no significant genetic differences detectable among strains within the same serotype (Fig. 1).

### Antimicrobial susceptibility of cefotaxime-resistant *Salmonella*

All the 79 cefotaxime-resistant foodborne *Salmonella* strains were found to be multidrug resistant (MDR) (Table S2). The rate of resistance to different classes of antimicrobial drugs is as follows: β-lactams: ampicillin (98.73%), ceftriaxone (100.00%); fluoroquinolones: ciprofloxacin (24.05%), nalidixic acid (49.37%); aminoglycosides: amikacin (5.06%), kanamycin (45.57%); macrolides: azithromycin (10.13%); tetracyclines: tetracycline (96.20%); phenicols: chloramphenicol (73.42%); and folate pathway inhibitors: trimethoprim-sulfamethoxazole (94.94%). Resistance to meropenem was not detectable. Among the cefotaxime-resistant *Salmonella* strains, the rate of resistance to ciprofloxacin was relatively high (> 30%) during the study period, except for the year 2016, when a resistance rate of 17.86% was recorded. The chicken-borne *Salmonella* strains exhibited higher rate of resistance to nearly all the antimicrobial drugs than strains recovered from pork, especially the key antimicrobial drugs for the treatment of *Salmonella* infections, namely, ciprofloxacin (52.94% vs 16.95%) and azithromycin (23.53% vs 6.78%); tetracycline was an exception. In addition, only chicken-borne *Salmonella* strains were resistant to amikacin (Table 3).

**TABLE 3:** The rate of antimicrobial resistance among cefotaxime-resistant foodborne *Salmonella* strains^a^

	No (%) of CTX^r^ isolates (%) in					No (%) of CTX^r^ isolates (%) in			
Antimicrobial drugs	Overall (*n* = 79)	2014 (*n* = 3)	2015 (*n* = 7)	2016 (*n* = 56)	2017 (*n* = 13)	Pork (*n* = 59)	Chicken (*n* = 17)	Beef (*n*=2)	Shrimp (*n* = 1)
AMP^a^	78 (98.73)	2 (67.67)	7 (100)	56 (100)	13 (100)	58 (98.31)	17 (100)	2 (100)	1 (100)
CTX	79 (100)	3 (100)	7 (100)	56 (100)	13 (100)	59 (100)	17 (100)	2 (100)	1 (100)
CRO	79 (100)	3 (100)	7 (100)	56 (100)	13 (100)	59 (100)	17 (100)	2 (100)	1 (100)
MRP	0 (0)	0 (0)	0 (0)	0 (0)	0 (0)	0 (0)	0 (0)	0 (0)	0 (0)
CHL	58 (73.42)	3 (100)	4 (57.14)	41 (73.21)	10 (76.92)	43 (72.88)	13 (76.47)	2 (100)	0 (0)
SXT	75 (94.94)	3 (100)	7 (100)	54 (96.43)	11 (84.62)	55 (93.22)	17 (100)	2 (100)	1 (100)
TET	76 (96.20)	2 (67.67)	6 (85.71)	55 (98.21)	13 (100)	57 (96.61)	16 (94.12)	2 (100)	1 (100)
CIP	19 (24.05)	1 (33.33)	3 (42.86)	10 (17.86)	5 (38.46)	10 (16.95)	9 (52.94)	0 (0)	0 (0)
NAL	39 (49.37)	3 (100)	3 (42.86)	24 (42.86)	9 (69.23)	24 (40.68)	15 (88.24)	0 (0)	0 (0)
AMK	4 (5.06)	0 (0)	1 (14.29)	3 (5.36)	0 (0)	0 (0)	4 (23.53)	0 (0)	0 (0)
AZI	8 (10.13)	2 (67.67)	1 (14.29)	4 (7.14)	1 (7.69)	4 (6.78)	4 (23.53)	0 (0)	0 (0)
KAN	36 (45.57)	1 (33.33)	4 (57.14)	23 (41.07)	8 (61.51)	19 (32.20)	11 (64.71)	2 (100)	0 (0)

^a^ AMP, Ampicillin; CTX, Cefotaxime; CRO, Ceftriaxone; MRP, Meropenem; CHL, Chloramphenicol; SXT, Sulfamethoxazole/Trimethoprim; TET, Tetracycline; CIP, Ciprofloxacin; NAL, Nalidixic acid; AMK, Amikacin; AZI, Azithromycin; KAN, Kanamycin.

### Genetic basis of cefotaxime resistance in *Salmonella*

DNA sequencing studies identified that seven CTX-M types β-lactamase genes were mostly responsible for cefotaxime resistance in the foodborne *Salmonella* strains, including one CTX-M-1 group gene: *bla*_CTX-M-55_; four CTX-M-9 group genes: *bla*_CTX-M-14_, *bla*_CTX-M-65_, *bla*_CTX-M-130_, and *bla*_CTX-M-27_; and one AmpC β-lactamase-encoding gene: *bla*_CMY-2_. The most common genes were *bla*_CTX-M-55_, *bla*_CTX-M-14,_ and *bla*_CTX-M-65_, accounting for 35.44%, 34.18%, and 22.78% of the test strains, respectively. In particular, strain SA1541 harbored both the CTX-M-1 group gene *bla*_CTX-M-55_ and the CTX-M-9 group gene *bla*_CTX-M-65_. The other five genes were less frequently found (< 3.00%). The prevalence rate of *bla*_CTX-M-55_ increased from 14.29% (1/7) in 2015 to 40.35% (23/57) in 2016 and then decreased slightly to 33.33% (4/12), whereas that of *bla*_CTX-M-14_ decreased from 71.43% (5/7) in 2015 to 33.33% (19/57) in 2016 and 25.00% (3/12) in 2017. Among the 47 cefotaxime-resistant *S.* Typhimurium strains, the most prevalent genes were *bla*_CTX-M-14_, *bla*_CTX-M-55,_ and *bla*_CTX-M-65_, accounting for 38.30%, 34.04%, and 25.53% of the test strains, respectively; 87.50% (*n* = 8) of *S.* Albany strains harbored *bla*_CTX-M-55,_ and 71.43% (*n* = 7) of *S.* Indiana strains carried *bla*_CTX-M-14_ (Table S3).

**Fig 2:**
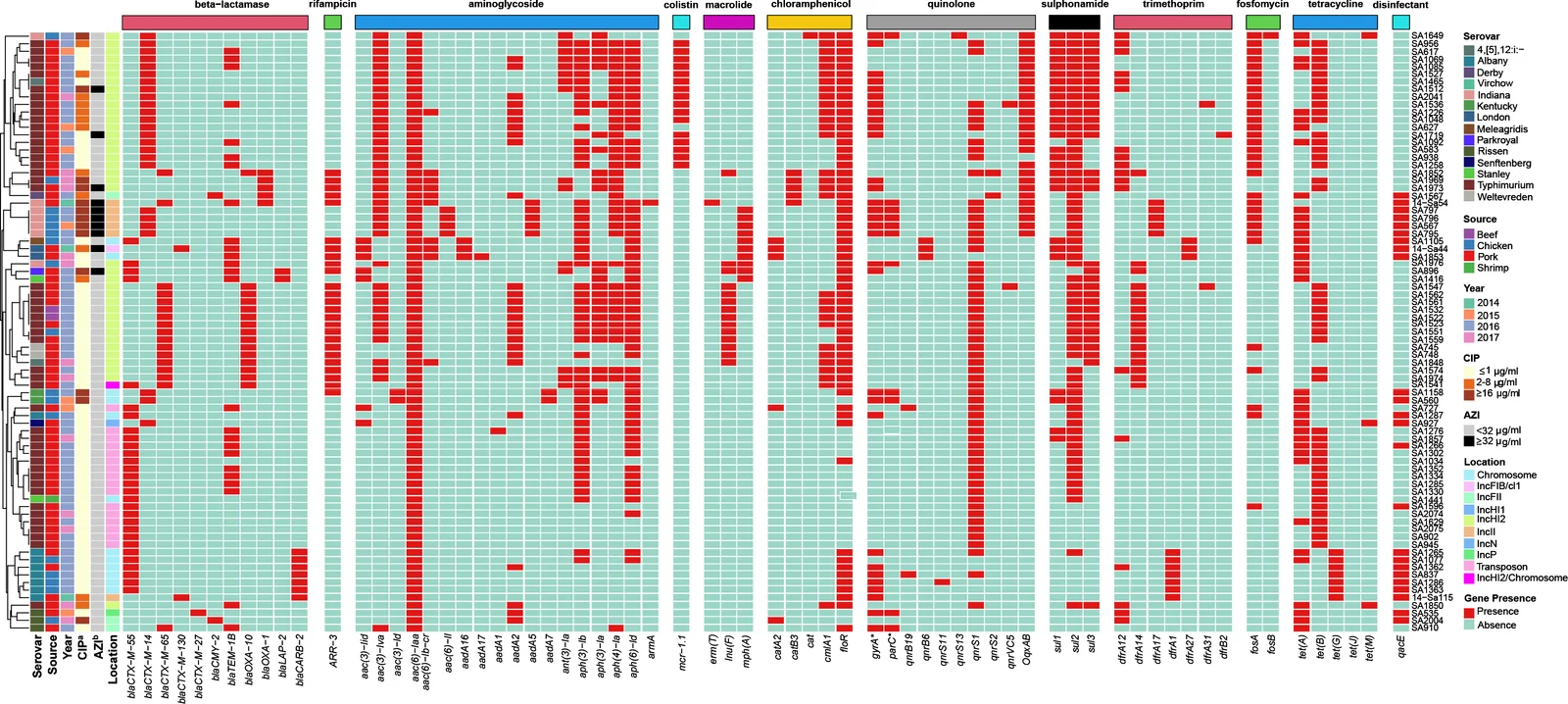
Heatmap of antibiotic resistance genes and plasmid replicon types among 79 cefotaxime-resistant foodborne *Salmonella* strains recovered during 2014–2017. The location means the distribution of cefotaxime-resistant genes. In the resistance genes-bearing region, red squares denote the carriage of specific resistance gene; a means ciprofloxacin MIC, b means azithromycin MIC, and * means the target mutation of *gyrA* or *parc* gene.

The test strains were also found to contain other β-lactamase genes such as those of the class A β-lactamase gene family [*bla*_TEM-1B_ (31.64%), *bla*_CARB-2_ (8.86%), and *bla*_LAP-2_ (2.53%)] and class D beta-lactamase gene family [*bla*_OXA-10_ (18.99%) and *bla*_OXA-1_ (6.33%)]. In addition, a variety of horizontally acquired antibiotic resistance genes were detectable; these include the rifampicin resistance gene *ARR-3*, the aminoglycoside resistance genes *aac(6’)-Iaa*, *aph (6)-Id*, *aph(3″)-Ib,* and *aac (3)-Iva*; the phenicol resistance genes *floR*, *drfA14,* and *drfA1*; the macrolide resistance genes *Inu(F)* and *mph(A)*; the fosfomycin resistance gene *fosA*; the colistin resistance gene *mcr-1.1*; the quinolone resistance genes *qnrS*, *qnrB*, *qnrVC,* and *oqxAB*; the sulfonamide resistance genes *sul1*, *sul2,* and *sul3*; and the tetracycline resistance genes *tet(B) tet(A)* (Fig. 2)

### Mobile genetic elements containing cefotaxime resistance genes

Majority of *bla*_CTX-M_ were plasmid-borne, and the rest were found in the chromosome and transposon. While *bla*_CMY-2_ gene was only detectable in plasmids. The BLAST results showed that all the contigs in 79 cefotaxime-resistant strains exhibited >99% identity and >96% coverage to structurally similar plasmids recorded in the NCBI database (Fig. S1 to S9), indicating the estimated replicon type and size of plasmids harbored cefotaxime resistance genes. Among the seven plasmid replicons that carried these genes, IncHI2 was predominant (50.63%, 40/79), followed by IncN (5.06%, 4/79) and IncFII (3.80%, 3/79). The detection frequency of IncFIB/I1, IncHI1, IncP, and IncI1 plasmids was 1.27% in each case (1/79). The rest of the cefotaxime resistance genes were located in the transposon and the chromosome (20.25% and 15.19%), respectively (Table 4).

**TABLE 4:** The brief of mobile genetic elements containing cefotaxime resistance genes

Type	Plasmid type	Size	Reference no.	β-lactamase	Genetic structure	No.	Conjugation efficiency
1	IncFIB/I1	202,750	MH430881.1	CTX-M-130	IS*Ecp1-bla*_CTX-M-130_-IS*903B*	1	NA
2.1	IncFII	186,499	CP047572.1	CTX-M-65	*bla*_CTX-M-65_-IS*903B*-IS*26*	1	1.31 × 10^−6^ − 9.23 × 10^−5^
2.2	IncFII	96,413	CP080258.1	CMY-2	IS*1294-bla*_CMY-2_	2	NA
3	IncHI1	244,597	AP020333.1	CTX-M-14	IS*Ecp1-bla*_CTX-M-14_-IS*903B*	1	NA
4.1	IncHI2	247,705	CP035918.1	CTX-M-14	IS*26-bla*_CTX-M-14_-*fosA*-IS*26*	20	1.49 × 10^−5^− 9.85 × 10^−5^
4.2	IncHI2	228,062	CP043951.1	CTX-M-65	IS26-*bla*_CTX-M-65_-IS*903B*	17	8.44 × 10^−5^− 1.09 × 10^−4^
4.3	IncHI2	236,068	MN539017.1	CTX-M-55	IS*26-bla*_LAP-2_-*bla*_TEM-1b_-Tn*2*-IS*Ecp1-bla*_CTX-M-55_-*orf*-Tn*2*-IS*2-qnrS1*-IS*26*	3	NA
5	IncN	85,943	CP047128.1	CTX-M-14	IS*26-bla*_CTX-M-14_-IS*903B*	4	NA
6	IncP	62,620	OP328419	CTX-M-27	IS*Ecp1-bla_CTX-M-27_*-IS*903B*	1	6.79 × 10^−6^ − 9.6 × 10^−5^
7	InclI	91,411	MH430883.1	CTX-M-130	*bla*_CTX-M-130_-IS*903B*	1	NA
8	Transposon	8,993	MN619286.1	CTX-M-55	IS*26*-IS*Ecp1-bla*_CTX-M-55_-*orf*-Tn*2*-IS*2-qnrS1*-IS*26*	16	NA
9.1	Chromosome			CTX-M-14		2	NA
9.2	Chromosome			CTX-M-55		9	NA
9.3	Chromosome			CTX-M-65		1	NA

On the contrary, 57.1% (16/28) of *bla*_CTX-M-55_ was found located in a chromosomal IS*26*-mediated composite transposon (GenBank accession number: MN619286.1) previously reported to be recovered from *Salmonella* strains in China (27); 10.7% of *bla*_CTX-M-55_ was located in IncHI2 plasmids and exhibited a high degree of sequence similarity to the pOYZ4 plasmid (GenBank accession number: MN539018.1) recovered from foodborne *Salmonella* strains.

About 74.1% (20/27) of *bla*_CTX-M-14_ was found located in IncHI2 plasmids, which are structurally highly similar (>99% identity) to the pLS44712-MCR plasmid (GenBank accession number: CP035918.1) recovered from clinical *Salmonella* strains in China; 14.8% of *bla*_CTX-M-14_ was carried by IncN plasmids that exhibited high-level sequence identity (>99%) to the pT-HNK130-3 plasmid (GenBank accession number: CP047128.1) recovered from *E. coli* strains in China; and 3.7% of *bla*_CTX-M-14_ was located in a IncHI1 plasmid that exhibited a high degree of sequence identity to the plasmid pSESen3709_1 (GenBank accession number: AP020333.1), which was recovered from *Salmonella* strains in Japan.

**Fig 3:**
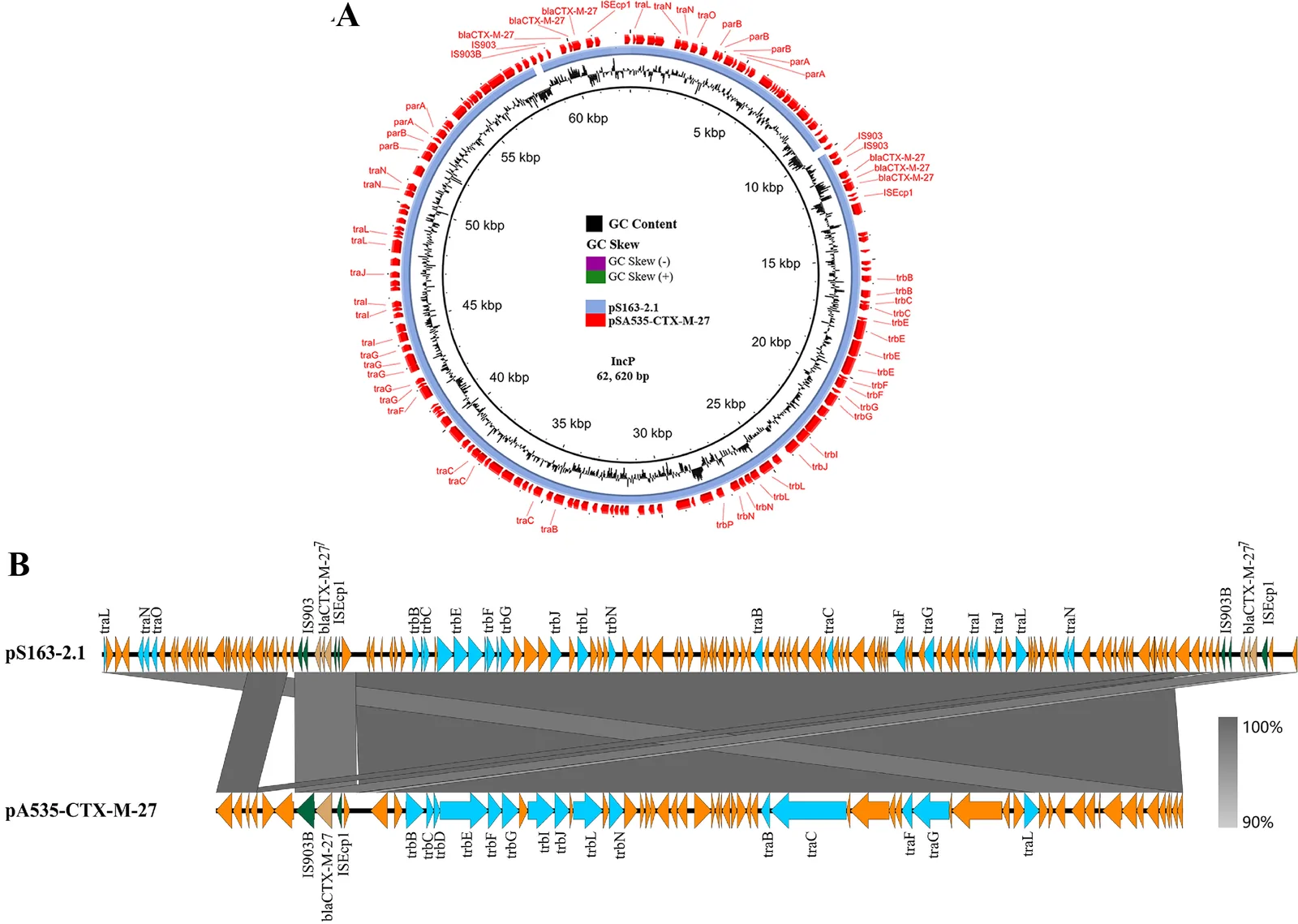
Alignment of pSA535-CTX-M-27 recovered from foodborne *Salmonella* strains in this study with structurally similar plasmids by BRIG and Easyfig. (**A**) Plasmid pSA535-CTX-M-27 exhibits the highest degree of similarity (98% coverage and 99.37% identity) to plasmid pS163-2.1 (GenBank accession number CP058710.1) in the NCBI database. (**B**) Alignment of plasmid pSA535-CTX-M-27 with plasmid pS163-2.1 by Easyfig. Brown arrows represent antibiotic-resistant genes; green depicts IS elements; and blue arrows are responsible for conjugative transfer genes.

About 89.5% (17/19) of *bla*_CTX-M-65_ was located in a IncHI2 plasmid, the structure of which was highly similar (>99% identity) to plasmid pST95-32-1 (GenBank accession number: CP043951.1), which was recovered from *E. coli* strains in China; 5.3% of *bla*_CTX-M-65_ was located in a IncFII plasmid that exhibited a high degree of sequence identity to plasmid p2EC1-1 (GenBank accession number: CP047572.1), which was recovered from a *E. coli* strain in Singapore. The *bla*_CTX-M-130_ harbored by a *Salmonella* London strain, 14-Sa44, was found located in a IncI1 plasmid pSA44-CRO (GenBank accession number: MH430883.1) and a fusion IncFIB/I1 plasmid pSa44-CIP-CRO (GenBank accession number: MH430881.1) reported previously (28). The *bla*_CTX-M-130_ was also detectable in a pSA44-CRO-like IncI1 plasmid in *Salmonella.* Albany strain 14-Sa115. The nanopore sequencing data depicted carriage of a unique *bla*_CTX-M-27_ in a 62,602 bp IncP plasmid named pSA535-CTX-M-27 (GenBank accession number: OP328419). The 62,602 bp IncP plasmid exhibited a high degree of similarity (98% coverage and 99.37% identity) to a 50,634 bp IncP plasmid known as pS163-2.1 (GenBank accession number CP058710.1). IncP plasmid pS163-2.1 was recovered from animal sourced *E. coli* in Guangzhou (Fig. 3A). Compared to pSA163-2.1, pSA535-CTX-M-27 contained more *tra* genes, including *traN*, *traI*, and *traJ*. Both plasmids harbored *traB*, *traC*, *traF*, *traG,* and *traL*, as well as a *bla*_CTX-M-27_ flanked by IS*Ecp1* and IS*903B*, but not other antibiotic resistance genes (Fig. 3B). Two *bla*_CMY-2_ were found located in a IncFII plasmid that was structurally similar to the pSJC33-3 plasmid (GenBank accession number: CP080258.1) recoverable from a *E. coli* strain in China.

**Fig 4:**
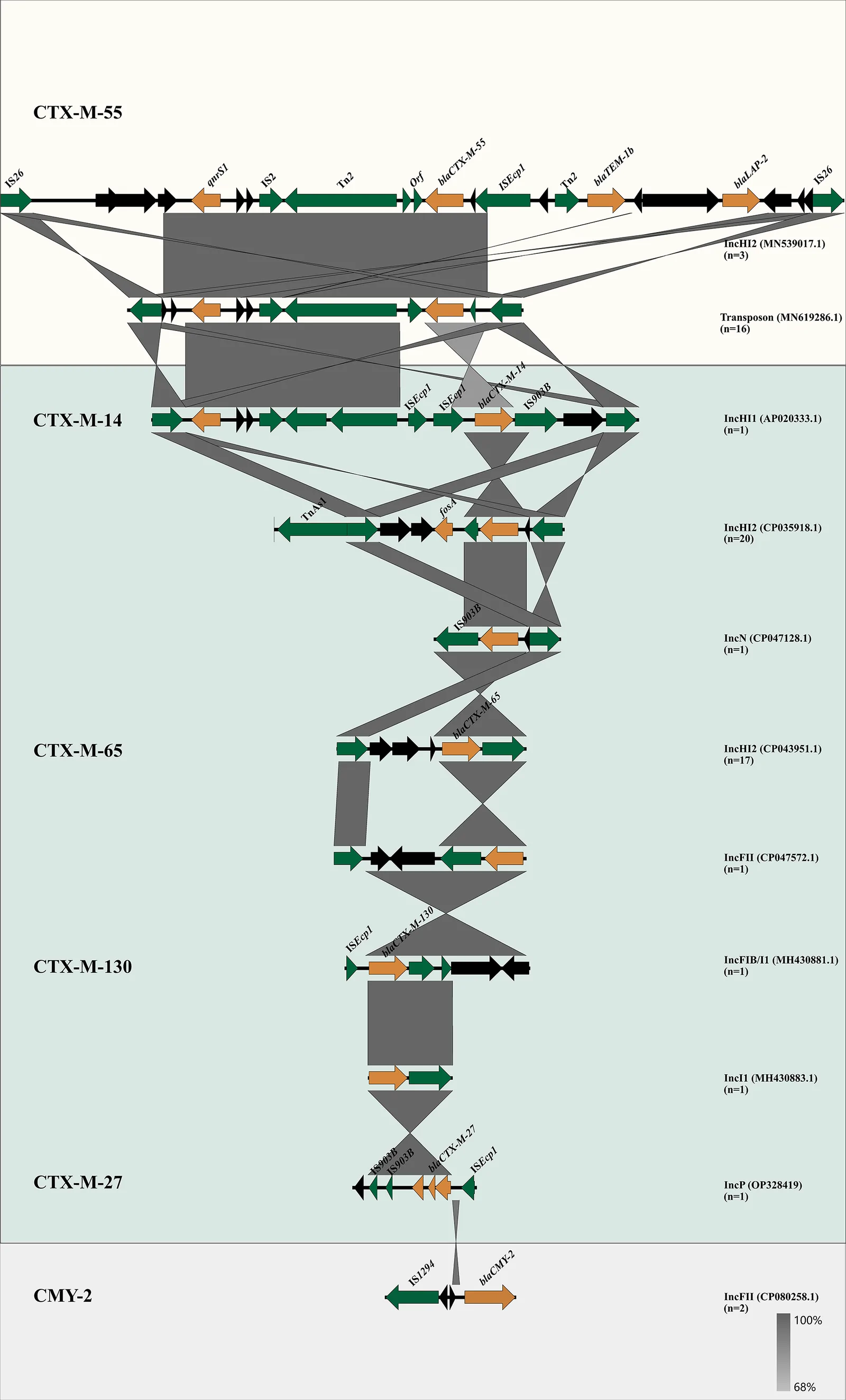
Genetic context of cefotaxime resistance genes located in plasmids and transposon. Yellow region represents CTX-M-1 group, green region represents the CTX-M-9 group, and gray region represents CMY-2. Brown arrows represent cefotaxime resistance genes, green arrows represent mobile genetic elements, and black arrows represent unrelated genes encoding unknown function. Plasmid replicon types and number, and accession numbers of the most similar plasmids of various gene arrays are depicted on the right.

Analysis of the genetic environment of the resistance genes in the test strains showed that IS*26*-ESBLs-IS*26* was the most prevalent genetic structure, followed by IS*26*-ESBLs-IS*903B* and IS*Ecp1*-ESBLs-IS*903B*, accounting for 49.37% and 26.58%, respectively. The *bla*_CTX-M-55_ was found in three different genetic structures, including the dominant chromosomal transposon-borne structure of IS*26*-IS*Ecp1-bla*_CTX-M-55_-*orf*-Tn*2*-IS*2-qnrS1*-IS*26*, a multidrug-resistance region of IS*26-bla*_LAP-2_-*bla*_TEM-1b_-Tn*2*-IS*Ecp1-bla*_CTX-M-55_-*orf*-Tn*2*-IS*2-qnrS1*-IS*26* in the IncHI2 plasmid and the chromosomal IS*Ecp1-bla*_CTX-M-55_ element. The most prevalent structure harboring *bla*_CTX-M-14_ was IS*26-bla*_CTX-M-14_-*fosA*-IS*26* in the IncHI2 plasmid, followed by IS*26-bla*_CTX-M-14_-IS*903B* in the IncN plasmids, IS*Ecp1-bla*_CTX-M-14_-IS*903B* in the IncHI1 plasmid, and IS*Ecp1-bla*_CTX-M-14_ in the chromosome. The *bla*_CTX-M-65_ existed in two different structures, which were the prevalent IS*26-bla*_CTX-M-65_-IS*903B* structure in the IncHI2 plasmid and the structure of *bla*_CTX-M-65_-IS*903B*-IS*26* in the IncFII plasmid, respectively. The genetic structure of *bla*_CTX-M-27_ and *bla*_CTX-M-130_ was identical, in which the resistance gene was flanked by the IS*Ecp1* and IS*903B* elements. Lastly, *bla*_CMY-2_ was found to be flanked by IS*1294* and located in the IncFII plasmid (Fig. 4).

### Conjugative transmission of mobile genetic elements containing cefotaxime resistance genes in *Salmonella*

Upon screening all the cefotaxime-resistant *Salmonella* by performing the conjugation experiments, we found that four of eight plasmid replicons were transferrable and could confer cefotaxime resistance to *E. coli J53.* The transferrable plasmid types were IncHI2 plasmid harboring *bla*_CTX-M-14_ with a conjugation frequency of 1.49 × 10^−5^ to 9.85 × 10^−5^, IncP plasmid containing *bla*_CTX-M-27_ with a frequency 6.79 × 10^−6^ to 9.6 × 10^−5^, and *bla*_CTX-M-65_ bearing IncHI2 and IncFII plasmids with a frequency 8.44 × 10^−5^ to 1.09 × 10^−4^ and 1.31 × 10^−6^ to 9.23 × 10^−5^, respectively. The cefotaxime MIC of the recipient strain *J53* increased from ≤0.0015 µg/mL to ≥16 µg/mL after acquiring these plasmids; 33.3% (9/27) of *bla*_CTX-M-55_, 7.4% (2/27) of the *bla*_CTX-M-14_, and 5.3% (1/19) of *bla*_CTX-M-65_ exhibited high-level sequence identity to the corresponding chromosomal elements recorded in the NCBI database. These genes were not transferable in conjugation experiments, further confirming that they were located in the chromosome.

## DISCUSSION

Cefotaxime resistance in *Salmonella* has become a serious public health issue as the choice of antibiotics in the treatment of *Salmonella* infection in cases where the organism invade beyond the gastrointestinal tract has become severely limited. It is necessary to conduct surveillance on the prevalence of cefotaxime-resistant foodborne *Salmonella* strains and investigate the underlying resistance and transmission mechanisms of such strains in order to devise effective measures to control the problem. In this study, we investigated the prevalence, antibiotic resistance phenotypes, and resistance and transmission mechanisms of cefotaxime-resistant *Salmonella* strains recovered from retail meat products in Shenzhen, China, during 2014–2017.

The overall isolation rate of cefotaxime-resistant foodborne *Salmonella* was 7.6%, and the rate recorded in individual year displayed an increasing trend from 2014 to 2017, which is similar to the previous studies (29 – 32). As in the case of previous studies, pork and chicken were the main sources of cefotaxime-resistant *Salmonella* (33, 34). Unlike other reports in China, *S.* Typhimurium was the most prevalent serovar among the cefotaxime-resistant *Salmonella* strains tested in this study (32, 35). The other serovars, including *S.* Indiana, *S*. Rissen, and *S*. Kentucky, were much less common than *S.* Typhimurium. One important finding in this study is that all cefotaxime-resistant *Salmonella* strains exhibited multidrug resistance (>3 antimicrobial drugs). In particular, 24.05% and 10.13% of the cefotaxime-resistant *Salmonella* strains exhibited resistance to ciprofloxacin and azithromycin, respectively. These two agents are also commonly used to treat *Salmonella* infection. As much as 7.59% of the cefotaxime-resistant *Salmonella* strains were resistant to both ciprofloxacin and azithromycin. Infections caused by such strains would have very limited treatment options. Co-carriage of different antibiotic resistance elements in one plasmid enables multi-resistant *Salmonella* strains to be readily selected upon exposure to only a single antibiotic, resulting in rapid expansion in population size of such strains.

In this work, sequencing analysis shows that the CTX-M genes are the key genetic elements that encode cefotaxime resistance in *Salmonella* and that AmpC β-lactamase gene also plays a partial role. The two most prevalent CTX-M genes were *bla*_CTX-M-55_ and *bla*_CTX-M-14_, respectively, and this observation is consistent with the previous reports on *Salmonella* recovered from patients, animals, and food samples in China (6, 36). CTX-M-65 was rarely detected in meat products in China but was found to be harbored by 22.78% of cefotaxime-resistant strains in our study (37). Besides, CTX-M-65 usually existed in Indiana and Infantis but became popular among the Typhimurium strains and those of other serovars in our work, including 4,[5],12:i:-, London, Rissen, and Weltevreden. Notably, a strain was found to contain the rare combination of *bla*_CTX-M-55_ and *bla*_CTX-M-65_; this is the first time such gene combination is detectable in foodborne *Salmonella* strains in China, where contamination of food samples by cefotaxime-resistant *Salmonella* strains is common. AmpC β-lactamase gene *bla*_CMY-2_ was previously popular in many countries while relatively rarely found in China, which existed in human, animal, and food (8, 38, 39). CMY-2 was also detectable among the test strains. Recovery of a wide range of resistance genes among foodborne *Salmonella* strains suggests that multiple development routes are responsible for the development of cefotaxime resistance in such strains.

Sequencing analysis in this work revealed the genetic location and environment of ESBL genes in the foodborne *Salmonella* strains. Consistent with previous studies, our findings show that most of the CTX-M genes are located in various plasmids and a chromosomal transposon and that such genes are often structurally accompanied with other antibiotic resistance genes. The most prevalent plasmid replicon that contained *bla*_CTX-M-14_ and *bla*_CTX-M-65_ was IncHI2, whereas the majority of the *bla*_CTX-M-55_ were located in a chromosomal IS*26*-mediated composite transposon. The structure IS*26*-IS*Ecp1-bla*_CTX-M-55_-*orf*-Tn*2*-IS*2-qnrS1*-IS*26* in chromosomal composite transposon was previously found in a chromosome of *Salmonella* Typhimurium strain S441, which was isolated from a human in Hangzhou, China (GenBank accession no. CP061122.1). Interestingly, all the strains that carried a transposon in this study were *Salmonella* Typhimurium. In view of the fact that the resistance gene was not transferable, we speculate that the transposon is located in the chromosome. The genetic structures of various CTX-M genes detected in this study were different from those described in previous reports. In this study, most of the *bla*_CTX-M-55_ were flanked by two IS*26* elements. Such structure was different from the IS*Ecp1-bla*_CTX-M-55_-IS*903B* structure observed in a previous study in Shanghai, China (36). The *bla*_CTX-M-14_ was located in various genetic structures and various types of plasmids; the predominant structure IS*26-bla*_CTX-M-14_-*fosA*-IS*26* was located in the IncHI2 plasmid, which was different from the IS*Ecp1-bla*_CTX-M-14_ structure reported in previous studies (40). The most prevalent structures of the most common cefotaxime genes, *bla*_CTX-M-55_ and *bla*_CTX-M-14_, were the same as that of the IS*26-bla_CTX-Ms_*-IS*26* structure. The IS*26-bla*_CTX-M-55_-IS*26* structure is located in 16 chromosomal transposons and 3 IncHI2 plasmids; the IS*26-bla*_CTX-M-14_-IS*26* structure was located in 20 IncHI2 plasmid. A previous study reported that a central fragment can constitute a translocatable unit (TU) by acquiring one adjacent IS, which confers the ability to undergo excision and transmission of antibiotic resistance genes from one plasmid to another or to the chromosome, and vice versa (41). Therefore, the IS*26-bla_CTX-Ms_*-IS*26* structure is supposed to be responsible for causing an increase in the prevalence of CTX-M-55 and CTX-M-14. Apart from the prevalent structure IS*26-bla*_CTX-M-65_-IS*903B*, *bla*_CTX-M-65_ was also found to be located downstream of IS*903B* and linked to the IS*26* element. The genetic structure of *bla*_CTX-M-27_ and *bla*_CTX-M-130_ was also the same as that described in previous findings, in which the genes were flanked by IS*Ecp1* and IS*903B* elements. The prevalent mobile elements linked to CTX-M genes were structurally different from those described in the previous reports, indicating that a wild range of mechanisms were responsible for the transmission of cefotaxime resistance-encoding elements among the foodborne *Salmonella* strains.

In summary, this study provides comprehensive insight into factors underlying the increasing prevalence of cefotaxime-resistant foodborne *Salmonella* strains in recent years. Acquisition of various ESBLs genes flanked by different mobile elements was found to be the major cause of increased prevalence of cefotaxime resistance in foodborne *Salmonella* strains. IncHI2 plasmids carrying the CTX-M-14 and CTX-M-65 genes and transposons harboring the CTX-M-55 gene were the dominant mobile genetic elements, whereas IS*26*-ESBLs-IS*26* and IS*26*-ESBLs-IS*903B* were the most common genetic structures. Importantly, all CTX-M-bearing plasmids were found to contain other antimicrobial resistance genes. To prevent the development of cefotaxime resistance, overuse and misuse of antimicrobial drugs that would select cefotaxime-resistant bacteria should be avoided to minimize selection and propagation of multidrug-resistant organisms. Surveillance of cefotaxime prescription criteria and treatment outcome should be performed to facilitate design of more appropriate cefotaxime usage guidelines to enhance treatment effectiveness and minimize resistance selection.

## Data Availability

All 79 cefotaxime-resistant Salmonella sequencing data have been deposited in GenBank under BioProject accession number PRJNA682289, and accession numbers have been listed in supplemental table S1. GenBank accession number OP328419 has been assigned to pSA535-CTX-M-27.

## SUPPLEMENTAL MATERIAL

Supplementary figures and tables spectrum.04886-22-s0001.docx Supplementary figures and tables

ASM does not own the copyrights to Supplemental Material that may be linked to, or accessed through, an article. The authors have granted ASM a non-exclusive, world-wide license to publish the Supplemental Material files. Please contact the corresponding author directly for reuse.
