# Supplementary material for: Prevalence and molecular characterization of cefotaxime-resistant Salmonella strains recovered from retail meat samples in Shenzhen, China, during 2014–2017
Source: Microbiol Spectr. 2023 Aug 24;11(5):e04886-22. doi: 10.1128/spectrum.04886-22 (PMC10580925; doi:10.1128/spectrum.04886-22)
Supplement: Supplementary figures and tables [file spectrum.04886-22-s0001.docx]

Supplementary Materials

**Prevalence and molecular characterization of cefotaxime resistant *Salmonella* strains recovered from retail meat samples in Shenzhen, China during 2014–2017**

Chen Yang^1,2^, Kaichao Chen^2, 3^, Lianwei Ye^1,2^, Heng Heng^1,2^, Xuemei Yang^2,3^, Edward Wai chi Chan^2^, Sheng Chen^2,3^*

Runing title: Cefotaxime-resistant foodborne *Salmonella* strains

^1^Department of Infectious Diseases and Public Health, Jockey Club College of Veterinary Medicine and Life Sciences, City University of Hong Kong, Kowloon, Hong Kong

^2^State Key Lab of Chemical Biology and Drug Discovery and the Department of Food Science and Nutrition, The Hong Kong Polytechnic University, Hung Hom, China

^3^Shenzhen Key Lab for Biological Safety Control, The Hong Kong Polytechnic University Shenzhen Research Institute, Shenzhen, China

Corresponding author: Sheng Chen, State Key Lab of Chemical Biology and Drug Discovery and the Department of Food Science and Nutrition, The Hong Kong Polytechnic University; Email: sheng.chen@polyu.edu.hk.

**Supplementary Table S1. Prevalence of cefotaxime resistant *Salmonella* strains in Shenzhen, China during the period 2014-2017**

|  | **No. of food sample and isolates** | | | | | | | | | |  |
| --- | --- | --- | --- | --- | --- | --- | --- | --- | --- | --- | --- |
|  | **Total** | | **Pork** | | **Chicken** | | **Beef** | | **Shrimp** | |  |
| **Year** | **Samples** | **Isolates** | **Samples** | **Isolates** | **Samples** | **Isolates** | **Samples** | **Isolates** | **Samples** | **Isolates** | **No. of CTX^r^ isolates (%)** |
| 2014 | 331 | 157 | 280 | 134 | 51 | 23 | None | None | None | None | 3 (1.9) |
| 2015 | 1012 | 286 | 567 | 192 | 170 | 70 | 99 | 16 | 176 | 8 | 7 (2.4) |
| 2016 | 1070 | 452 | 535 | 264 | 213 | 127 | 131 | 47 | 191 | 14 | 56 (12.4) |
| 2017 | 562 | 143 | 329 | 104 | 95 | 37 | 64 | 2 | 74 | 0 | 13 (9.1) |
| Total | 2975 | 1038 | 1711 | 694 | 529 | 257 | 294 | 65 | 441 | 22 | 79 (7.6) |

CTX^r^: Cefotaxime resistant.

**Supplementary Table S2. MIC profiles and accession number of 79 cefotaxime resistant foodborne *Salmonella* isolates.**

|  | **Accession number** | **MIC (μg/ml)** | | | | | | | | | | | |
| --- | --- | --- | --- | --- | --- | --- | --- | --- | --- | --- | --- | --- | --- |
| **Strain ID** |  | **AMP** | **CTX** | **CRO** | **MRP** | **CIP** | **NAL** | **AZI** | **CHL** | **SXT** | **TET** | **AMK** | **KAN** |
| 14-Sa44 | JAELQH000000000 | 0.5 | >16 | >16 | 0.06 | 2 | 64 | >32 | >64 | >32 | >32 | 4 | 64 |
| 14-Sa54 | JAELQC000000000 | >128 | >16 | >16 | 0.03 | >16 | >64 | >32 | >64 | 32 | 0.5 | 1 | 16 |
| 14-Sa115 | JAELOZ000000000 | >64 | >16 | >16 | 0.12 | 2 | >64 | 2 | >64 | >32 | 32 | 4 | 1 |
| SA535 | JANFQK000000000 | >64 | >16 | >16 | 0.06 | 0.03 | 4 | <0.25 | 2 | >32 | >32 | 4 | 4 |
| SA560 | JAELJO000000000 | >64 | >16 | >16 | 0.03 | 16 | >64 | 1 | 2 | 4 | >32 | 4 | >64 |
| SA567 | JAELJN000000000 | >64 | >16 | >16 | 0.03 | >16 | >64 | 32 | >64 | >32 | 32 | 128 | >64 |
| SA583 | JAELJI000000000 | >64 | 16 | >16 | 0.03 | 0.12 | 16 | <0.25 | 8 | >32 | >32 | 2 | >64 |
| SA617 | JAELIY000000000 | >64 | >16 | >16 | 0.03 | 0.25 | 16 | 0.5 | >64 | >32 | >32 | 4 | >64 |
| SA627 | JAELAP000000000 | >64 | >16 | >16 | 0.03 | 4 | >64 | 1 | >64 | >32 | 1 | 4 | 8 |
| SA727 | JAEKZQ000000000 | >64 | >16 | >16 | 0.06 | 1 | 16 | 1 | 64 | 4 | 32 | 2 | 8 |
| SA745 | JAEKZL000000000 | >64 | >16 | >16 | 0.06 | 1 | 16 | 1 | 64 | >32 | 32 | 2 | 4 |
| SA748 | JAEKZJ000000000 | >64 | >16 | >16 | 0.06 | 1 | 16 | 1 | 64 | >32 | 32 | 2 | 2 |
| SA795 | JAEKZA000000000 | >64 | >16 | >16 | 0.12 | >16 | >64 | >32 | >64 | >32 | 32 | 64 | >128 |
| SA796 | JAEKYZ000000000 | >64 | >16 | >16 | 0.12 | >16 | >64 | >32 | 64 | >32 | 32 | >128 | >128 |
| SA797 | JAEKYY000000000 | >64 | >16 | >16 | 0.12 | >16 | >64 | >32 | >64 | >32 | 32 | >128 | >128 |
| SA837 | JAEKYL000000000 | >64 | >16 | >16 | 0.12 | 1 | >64 | 2 | >64 | >32 | 32 | 2 | 2 |
| SA896 | JAEKXQ000000000 | >64 | >16 | >16 | 0.06 | >16 | >64 | 32 | >64 | >32 | >32 | 1 | >128 |
| SA902 | JAEKXP000000000 | >64 | >16 | >16 | 0.12 | 0.5 | 8 | 1 | 2 | 16 | >32 | 2 | 4 |
| SA910 | JANFQJ000000000 | >64 | >16 | >16 | 0.12 | 0.03 | 4 | 1 | >64 | 8 | >32 | 4 | 16 |
| SA927 | JAEKXI000000000 | >64 | >16 | >16 | 0.06 | 1 | 8 | 1 | 32 | >32 | >32 | 4 | 16 |
| SA938 | JAEKXE000000000 | >64 | >16 | >16 | 0.06 | 0.12 | 8 | 4 | >64 | >32 | >32 | 2 | 4 |
| SA945 | JAEKXD000000000 | >64 | >16 | >16 | 0.06 | 0.5 | 4 | 1 | 2 | 2 | >32 | 2 | 4 |
| SA956 | JAEKXC000000000 | >64 | >16 | >16 | 0.06 | 4 | >64 | 2 | >64 | >32 | >32 | 2 | >128 |
| SA1034 | JAEKWE000000000 | >64 | >16 | >16 | 0.06 | 1 | 4 | 1 | 2 | 8 | >32 | 2 | 4 |
| SA1048 | JAEKVX000000000 | >64 | >16 | >16 | 0.06 | 2 | >64 | 1 | >64 | >32 | >32 | 4 | 8 |
| SA1069 | JAEKVR000000000 | >64 | 16 | >16 | 0.06 | 0.03 | 8 | 1 | 64 | 16 | >32 | 2 | 128 |
| SA1077 | JAEKVK000000000 | >64 | >16 | >16 | 0.06 | 1 | >64 | 2 | 64 | >32 | >32 | 2 | 2 |
| SA1085 | JAEKVH000000000 | >64 | >16 | >16 | 0.06 | 0.06 | 8 | 1 | 64 | >32 | >32 | 2 | >128 |
| SA1092 | JAEKVF000000000 | >64 | 16 | >16 | 0.06 | 0.06 | 8 | 1 | 64 | >32 | >32 | 2 | 4 |
| SA1105 | JAEKVA000000000 | >64 | >16 | >16 | 0.06 | 1 | 32 | 16 | >64 | >32 | 16 | 4 | 4 |
| SA1158 | JAEKUD000000000 | >64 | >16 | >16 | 0.06 | 16 | >64 | 0.5 | 2 | >32 | 32 | 4 | >128 |
| SA1226 | JAEKSW000000000 | >64 | >16 | >16 | 0.03 | 2 | >64 | 2 | >64 | >32 | >32 | 2 | 4 |
| SA1258 | JAEKSE000000000 | >64 | >16 | >16 | 0.06 | 0.12 | 32 | 0.5 | >64 | >32 | >32 | 4 | 4 |
| SA1265 | JAEKSC000000000 | >64 | >16 | >16 | 0.06 | 1 | >64 | 1 | 64 | >32 | 32 | 4 | 4 |
| SA1266 | JAEKSB000000000 | >64 | >16 | >16 | 0.06 | 0.5 | 4 | 0.5 | 1 | 8 | >32 | 4 | 4 |
| SA1276 | JAEKRU000000000 | >64 | >16 | >16 | 0.03 | 0.5 | 8 | 1 | 2 | 8 | >32 | 4 | 4 |
| SA1285 | JAEKRR000000000 | >64 | >16 | >16 | 0.12 | 0.5 | 8 | 1 | 2 | 8 | >32 | 4 | 4 |
| SA1286 | JAEKRQ000000000 | >64 | >16 | >16 | 0.06 | 1 | >64 | 1 | >64 | >32 | >32 | 4 | 4 |
| SA1287 | JAEKRP000000000 | >64 | >16 | >16 | 0.06 | 1 | >64 | 1 | 1 | 8 | 2 | 4 | 4 |
| SA1302 | JAEKRJ000000000 | >64 | >16 | >16 | 0.03 | 0.5 | <0.5 | 1 | 2 | 8 | >32 | 4 | 4 |
| SA1330 | JAEKQR000000000 | >64 | >16 | >16 | 0.03 | 0.5 | 8 | 1 | 2 | 16 | >32 | 2 | 4 |
| SA1334 | JAEKQQ000000000 | >64 | >16 | >16 | 0.03 | 0.5 | 8 | 1 | 2 | 32 | >32 | 4 | 4 |
| SA1352 | JAEKQG000000000 | >64 | >16 | >16 | <0.0075 | 0.5 | 8 | 1 | 2 | 32 | >32 | 4 | 4 |
| SA1362 | JAEKQC000000000 | >64 | >16 | >16 | 0.06 | 0.5 | >64 | 2 | >64 | >32 | 32 | 2 | 4 |
| SA1363 | JAEKQA000000000 | >64 | >16 | >16 | 0.06 | 1 | >64 | 2 | >64 | >32 | >32 | 4 | 4 |
| SA1416 | JAELIQ000000000 | >64 | >16 | >16 | 0.06 | 2 | 64 | 16 | >64 | 8 | >32 | 2 | >128 |
| SA1441 | JASXSS000000000 | >64 | >16 | >16 | 0.12 | 0.5 | 8 | 0.25 | 2 | 8 | >32 | 4 | 2 |
| SA1465 | JAELIA000000000 | >64 | >16 | >16 | 0.03 | 1 | >64 | 1 | >64 | >32 | >32 | 1 | 4 |
| SA1512 | JAELHS000000000 | >64 | >16 | >16 | 0.06 | >16 | >64 | 8 | >64 | >32 | >32 | 4 | >128 |
| SA1522 | JAELHQ000000000 | >64 | >16 | >16 | 0.06 | 1 | 16 | 1 | >64 | >32 | >32 | 4 | >128 |
| SA1523 | JAELHP000000000 | >64 | >16 | >16 | 0.03 | 1 | 16 | 0.5 | 64 | >32 | >32 | 1 | >128 |
| SA1527 | JAELHO000000000 | >64 | >16 | >16 | 0.06 | 2 | >64 | 2 | >64 | >32 | >32 | 2 | 2 |
| SA1532 | JAELHM000000000 | >64 | >16 | >16 | 0.03 | 1 | 16 | 2 | >64 | >32 | >32 | 1 | >128 |
| SA1536 | JAELHJ000000000 | >64 | >16 | >16 | 0.06 | 4 | >64 | 2 | >64 | 2 | >32 | 2 | >128 |
| SA1541 | JAELHG000000000 | >64 | >16 | >16 | 0.03 | 0.5 | 8 | 0.5 | 64 | >32 | 32 | 1 | 2 |
| SA1547 | JAELHE000000000 | >64 | >16 | >16 | 0.06 | 1 | 16 | 1 | >64 | >32 | >32 | 4 | >128 |
| SA1551 | JAELHC000000000 | >64 | >16 | >16 | 0.06 | 1 | 16 | 0.5 | 64 | >32 | >32 | 4 | >128 |
| SA1559 | JAELHA000000000 | >64 | >16 | >16 | 0.06 | 1 | 16 | 1 | >64 | >32 | >32 | 4 | >128 |
| SA1561 | JAELGZ000000000 | >64 | >16 | >16 | 0.06 | 1 | 16 | 0.5 | 64 | >32 | >32 | 2 | >128 |
| SA1562 | JAELGY000000000 | >64 | >16 | >16 | 0.06 | 1 | 16 | 1 | 64 | >32 | >32 | 2 | >128 |
| SA1567 | JAELGV000000000 | >64 | >16 | >16 | 0.12 | 8 | 32 | 0.5 | >64 | >32 | 32 | 8 | 128 |
| SA1574 | JAELGT000000000 | >64 | >16 | >16 | 0.06 | 1 | 16 | 1 | >64 | >32 | >32 | 2 | >128 |
| SA1596 | JAELGP000000000 | >64 | >16 | >16 | 0.06 | 0.5 | 8 | 0.5 | 1 | 32 | >32 | 2 | 8 |
| SA1629 | JAELGI000000000 | >64 | >16 | >16 | 0.12 | 0.5 | 8 | 1 | 2 | 16 | >32 | 2 | 4 |
| SA1649 | JAELGC000000000 | >64 | >16 | >16 | 0.06 | 16 | >64 | 0.5 | 64 | >32 | 32 | 2 | >128 |
| SA1719 | JAELFL000000000 | >64 | 8 | 16 | 0.03 | 1 | 8 | >32 | >64 | >32 | >32 | 4 | 128 |
| SA1848 | JAELEM000000000 | >64 | >16 | >16 | 0.03 | 1 | 4 | 0.5 | >64 | 2 | >32 | 2 | >128 |
| SA1850 | JAELEL000000000 | >64 | >16 | >16 | 0.03 | 2 | 8 | 8 | >64 | 2 | >32 | 4 | >128 |
| SA1852 | JAELEK000000000 | >64 | >16 | >16 | 0.03 | 8 | 32 | 0.5 | >64 | >32 | >32 | 8 | >128 |
| SA1853 | JAELEJ000000000 | >64 | >16 | >16 | 0.03 | 1 | >64 | 0.5 | >64 | >32 | >32 | 2 | 8 |
| SA1857 | JAELEH000000000 | >64 | >16 | >16 | 0.03 | 0.5 | 8 | 1 | 4 | 4 | >32 | 2 | 4 |
| SA1969 | JAELDD000000000 | >64 | >16 | >16 | 0.06 | >16 | >64 | 16 | >64 | >32 | >32 | 1 | >128 |
| SA1973 | JAELDB000000000 | >64 | >16 | >16 | 0.06 | >16 | >64 | 8 | >64 | >32 | >32 | 1 | >128 |
| SA1974 | JAELDA000000000 | >64 | >16 | >16 | 0.06 | 1 | 32 | 1 | >64 | >32 | >32 | 4 | >128 |
| SA1976 | JAELCY000000000 | >64 | >16 | >16 | 0.06 | 1 | 16 | 1 | 16 | >32 | >32 | 1 | >128 |
| SA2004 | JAELCN000000000 | >64 | >16 | >16 | 0.03 | >16 | >64 | 1 | >64 | >32 | 32 | 1 | >128 |
| SA2041 | JAELBR000000000 | >64 | >16 | >16 | 0.03 | 4 | 32 | 1 | >64 | >32 | >32 | 8 | 32 |
| SA2074 | JAELAW000000000 | >64 | >16 | >16 | 0.03 | 0.5 | >64 | 2 | 32 | 32 | >32 | 8 | 8 |
| SA2075 | JAELAV000000000 | >64 | >16 | >16 | 0.03 | 1 | >64 | 1 | 8 | >32 | >32 | 8 | 4 |

AMP, Ampicillin; CTX, Cefotaxime; CRO, Ceftriaxone; MRP: Meropenem; CIP, Ciprofloxacin; NAL, Nalidixic acid; AZI, Azithromycin; CHL, Chloramphenicol; SXT, Sulfamethoxazole/Trimethoprim; TET, Tetracycline; AMK, Amikacin; KAN, Kanamycin.

**Supplementary Table S3. Genotypes of cefotaxime resistant *Salmonella* strains**

|  | **Number of CTX^r^ isolates (%)** | | | |  |
| --- | --- | --- | --- | --- | --- |
| **Gene** | **2014** | **2015** | **2016** | **2017** | **Total** |
| *bla*_CTX-M-55_ | 0 (0.00) | 1 (14.29) | 23(40.35) | 4 (33.33) | 28 (35.44) |
| *bla*_CTX-M-14_ | 0 (0.00) | 5 (71.43) | 19 (33.33) | 3 (25.00) | 27 (34.18) |
| *bla*_CTX-M-65_ | 1 (33.33) | 0 (0.00) | 12 (22.81) | 4 (33.33) | 17 (22.78) |
| *bla*_CTX-M-130_ | 2 (67.67) | 0 (0.00) | 0 (0.00) | 0 (0.00) | 2 (2.53) |
| *bla*_CTX-M-27_ | 0 (0.00) | 1 (14.29) | 0 (0.00) | 0 (0.00) | 1 (1.27) |
| *bla*_CMY-2_ | 0 (0.00) | 0 (0.00) | 1 (1.75) | 1 (8.33) | 2 (2.53) |
| *bla*_CTX-M-55/65_ | 0 (0.00) | 0 (0.00) | 1 (1.75) | 0 (0.00) | 1 (1.27) |
| Total | 3 | 7 | 56 | 12 | 79 |

**
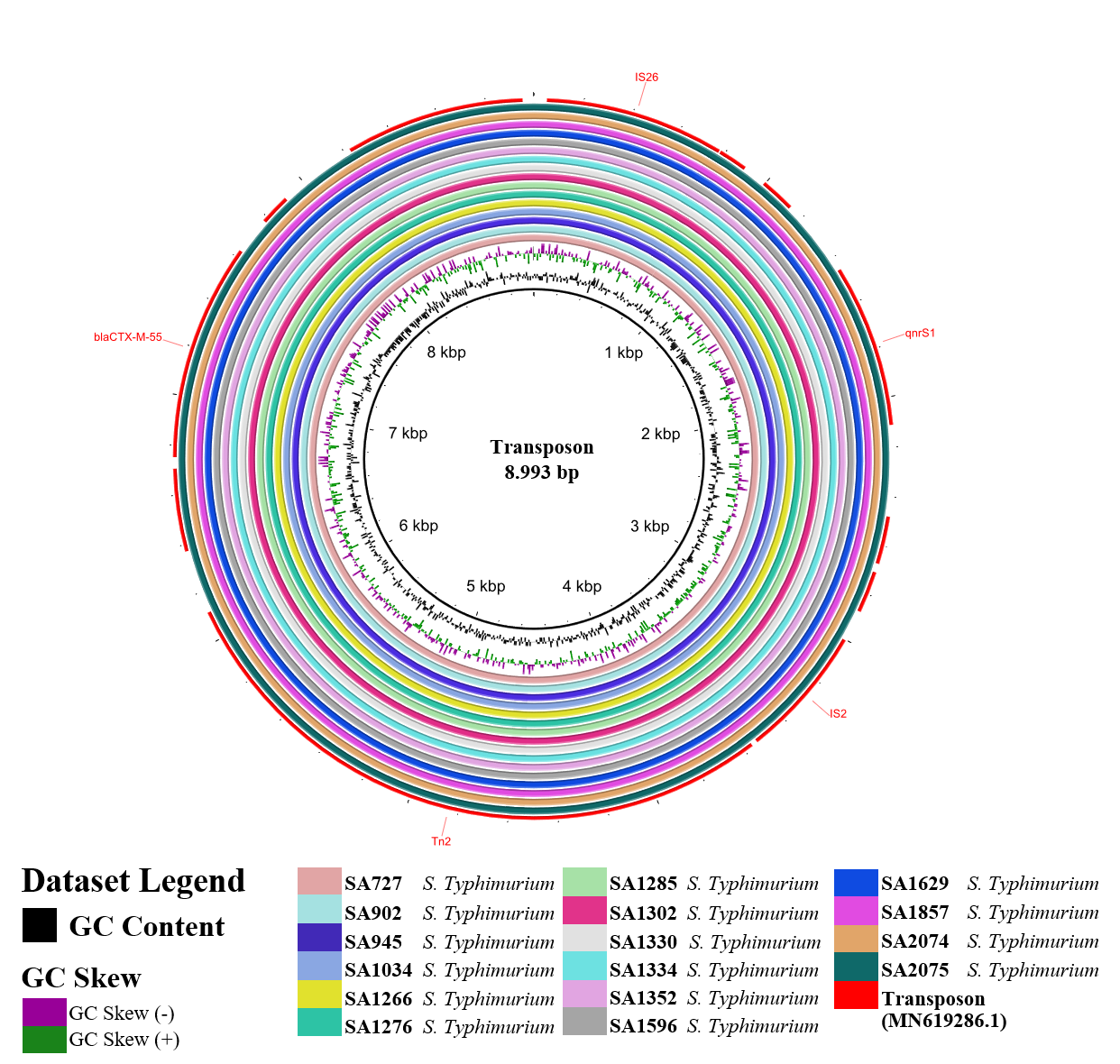
**

**Supplementary Figure S1. Alignment of *bla*_CTX-M-55_-bearing transposon from foodborne *Salmonella* in this study with a similar transposon by BLAST Ring Image Generator (BRIG).** Illumina contigs of 16 CTX-M-55-positive *Salmonella* show high similarity to the transposon (GenBank accession number MN619286.1) in the NCBI database.


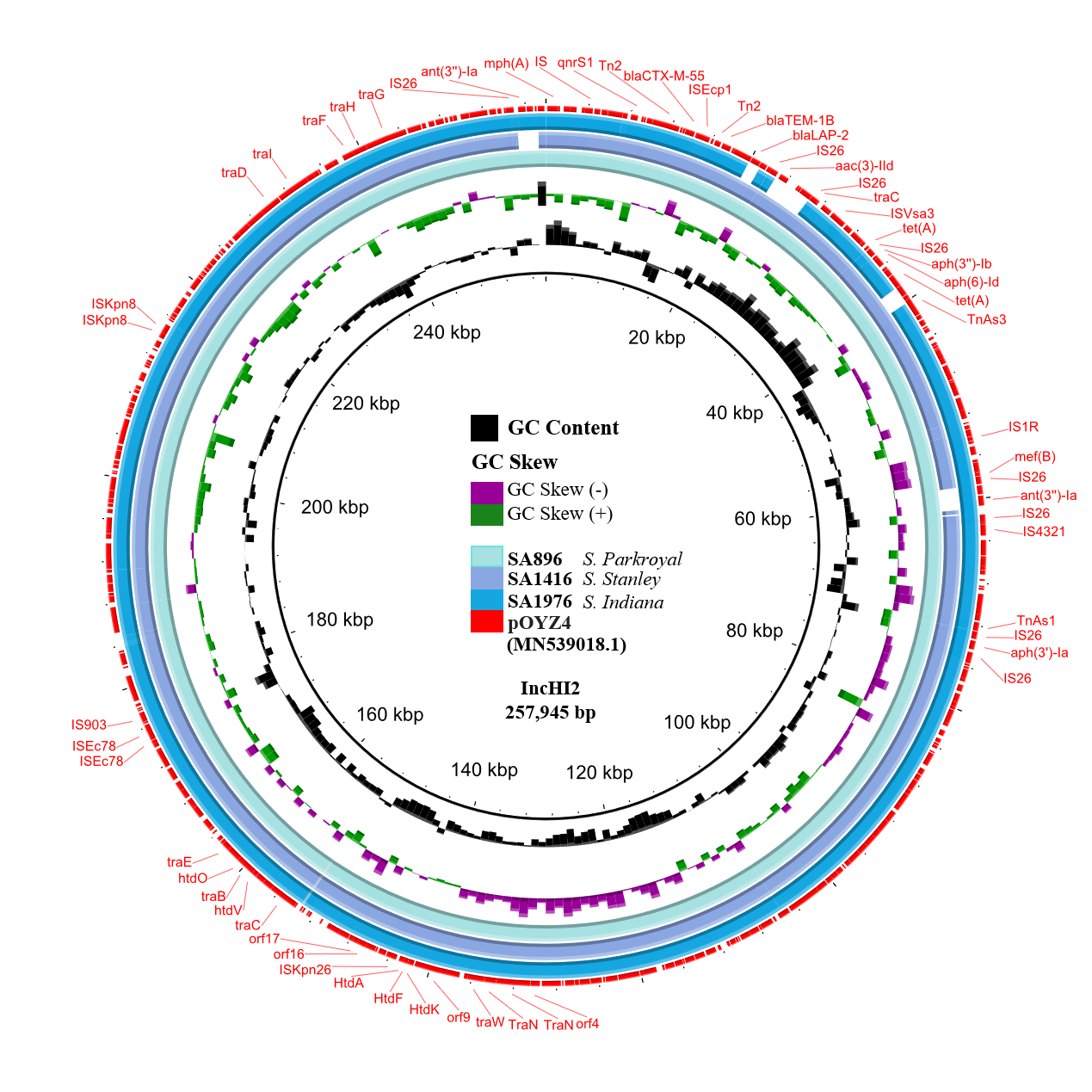


**Supplementary Figure S2. Alignment of *bla*_CTX-M-55_-bearing plasmids from foodborne *Salmonella* in this study with a similar IncHI2 plasmid by BRIG.** Illumina contigs of 3 CTX-M-55-bearing plasmids show high similarity to the IncHI2 plasmid pOYZ4 (GenBank accession number MN539018.1) in the NCBI database.


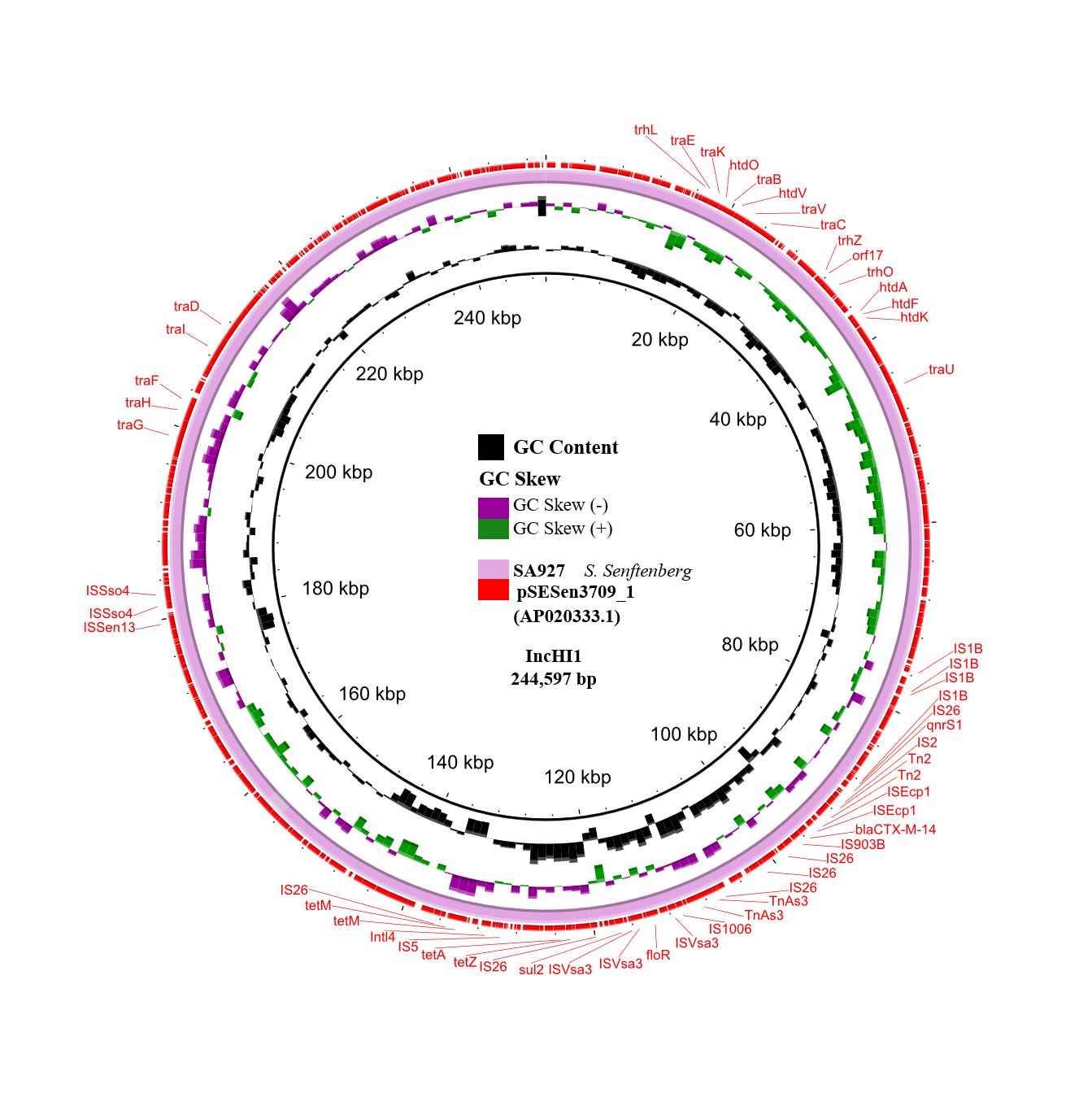


**Supplementary Figure S3. Alignment of a *bla*_CTX-M-14_-bearing plasmid from foodborne *Salmonella* in this study with a similar IncHI1 plasmid by BRIG.** Illumina contigs of a CTX-M-14-bearing plasmid show high similarity to the IncHI1 plasmid pSESen3709_1 (GenBank accession number AP020333.1) in the NCBI database.


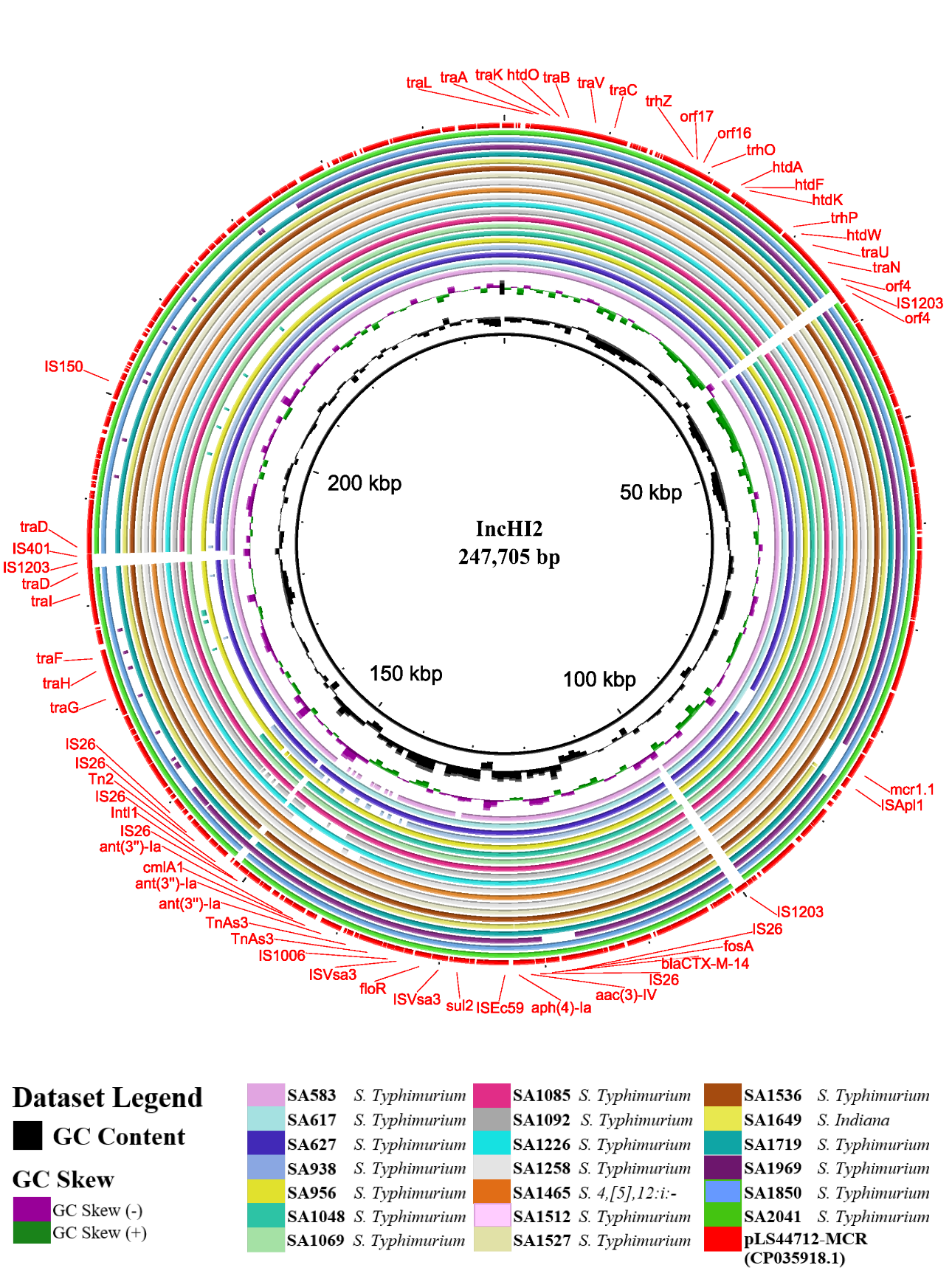


**Supplementary Figure S4. Alignment of *bla*_CTX-M-14_-bearing plasmids from foodborne *Salmonella* in this study with a similar IncHI2 plasmid by BRIG.** Illumina contigs of 19 CTX-M-14-bearing plasmids show high similarity to the IncHI2 plasmid pLS44712-MCR (GenBank accession number CP035918.1) in the NCBI database.


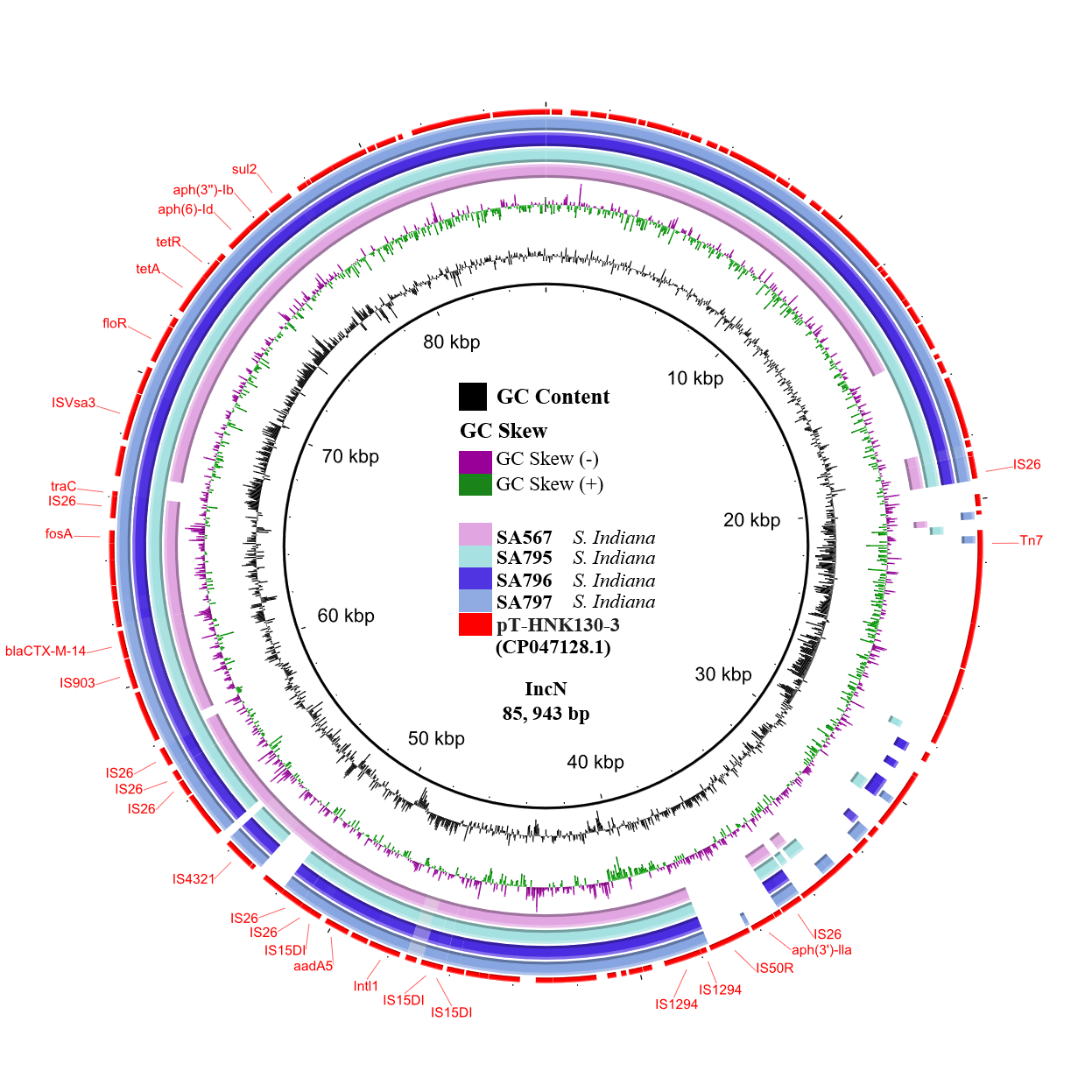


**Supplementary Figure S5. Alignment of *bla*_CTX-M-14_-bearing plasmids from foodborne *Salmonella* in this study with a similar IncN plasmid by BRIG.** Illumina contigs of 4 CTX-M-14-bearing plasmids show high similarity to the IncN plasmid pT-HNK130-3 (GenBank accession number CP047128.1) in the NCBI database.


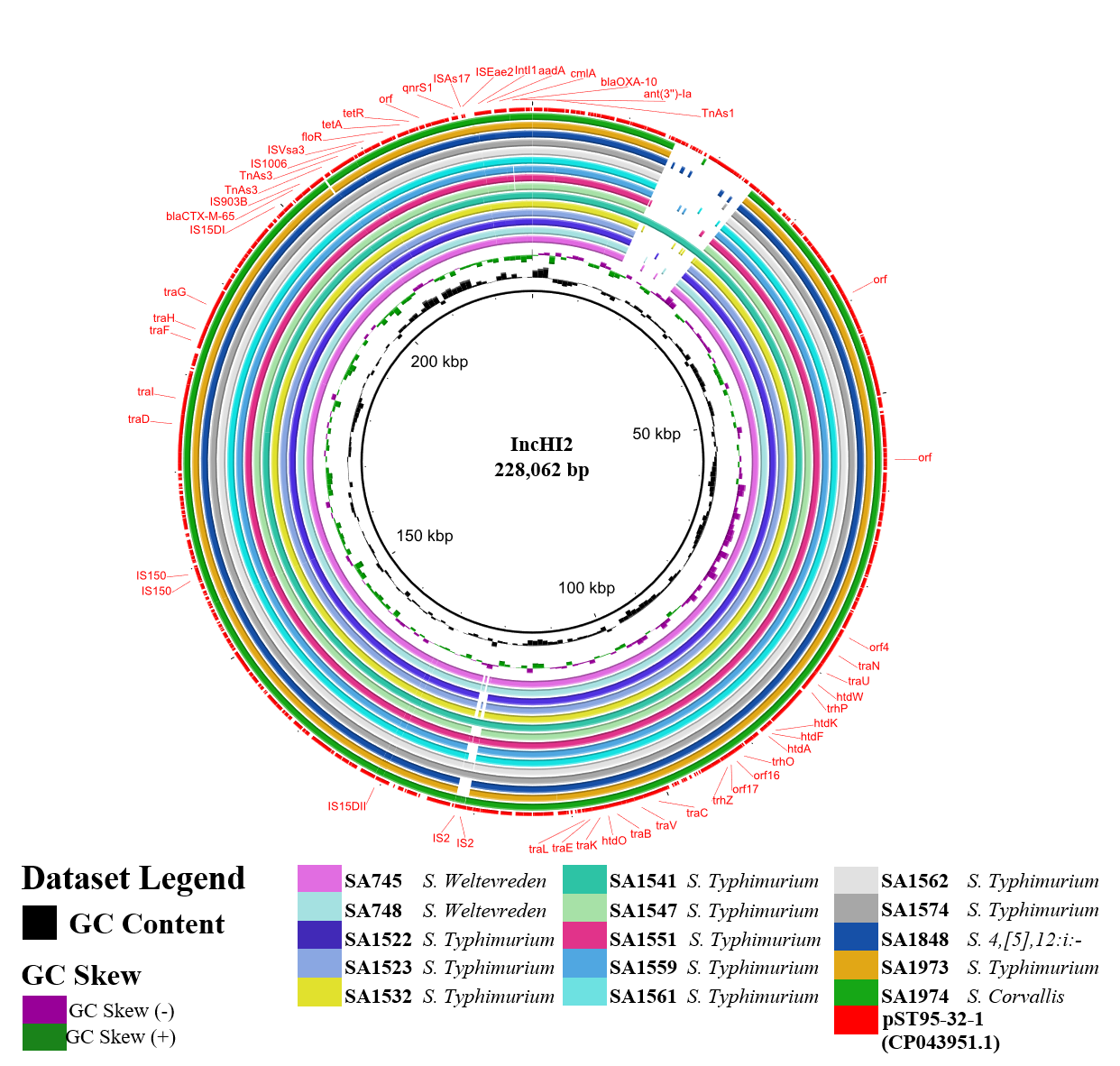


**Supplementary Figure S6. Alignment of *bla*_CTX-M-65_-bearing plasmids from foodborne *Salmonella* in this study with a similar IncHI2 plasmid by BRIG.** Illumina contigs of 16 CTX-M-65-bearing plasmids show high similarity to the IncHI2 plasmid pST95-32-1 (GenBank accession number CP043951.1) in the NCBI database.


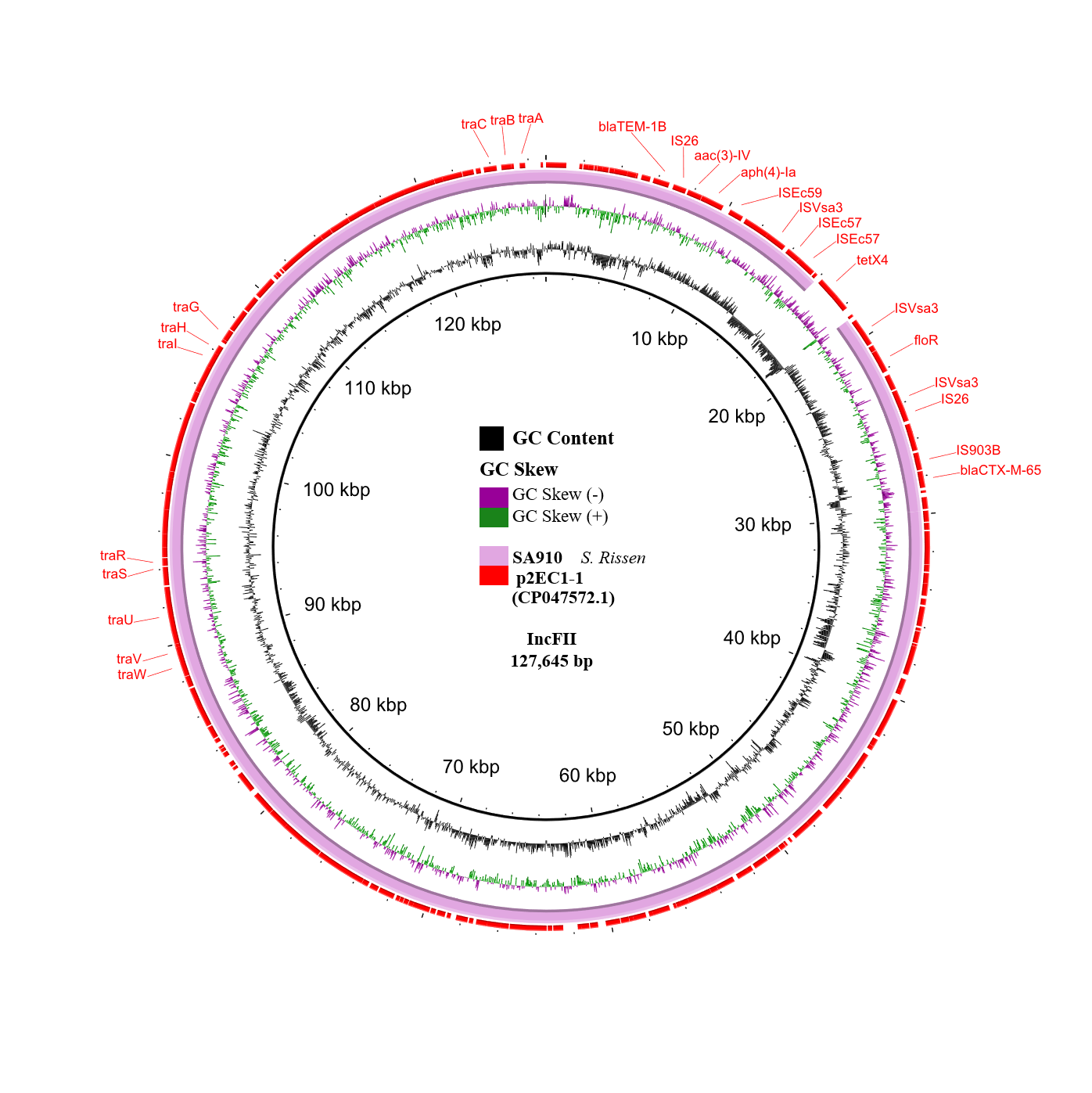


**Supplementary Figure S7. Alignment of a *bla*_CTX-M-65_-bearing plasmid from foodborne *Salmonella* in this study with a similar IncFII plasmid by BRIG.** Illumina contigs of a CTX-M-65-bearing plasmid show high similarity to the IncFII plasmid p2EC1-1 (GenBank accession number CP047572.1) in the NCBI database.


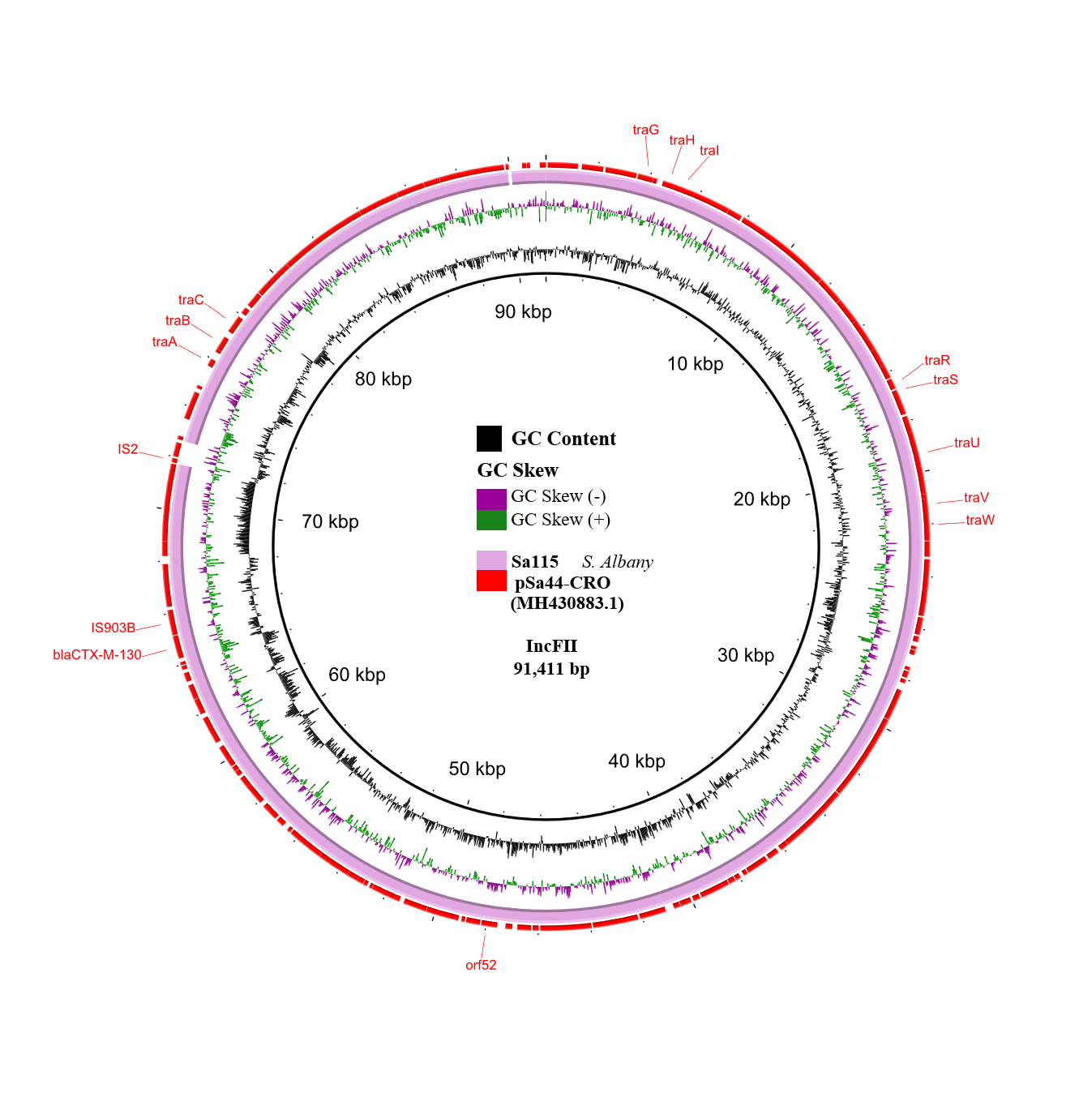


**Supplementary Figure S8. Alignment of a *bla*_CTX-M-130_-bearing plasmid from foodborne *Salmonella* in this study with a similar IncFII plasmid by BRIG.** Illumina contigs of a CTX-M-130-bearing plasmid show high similarity to the IncFII plasmid pSA44-CRO (GenBank accession number CP430883.1) in the NCBI database.


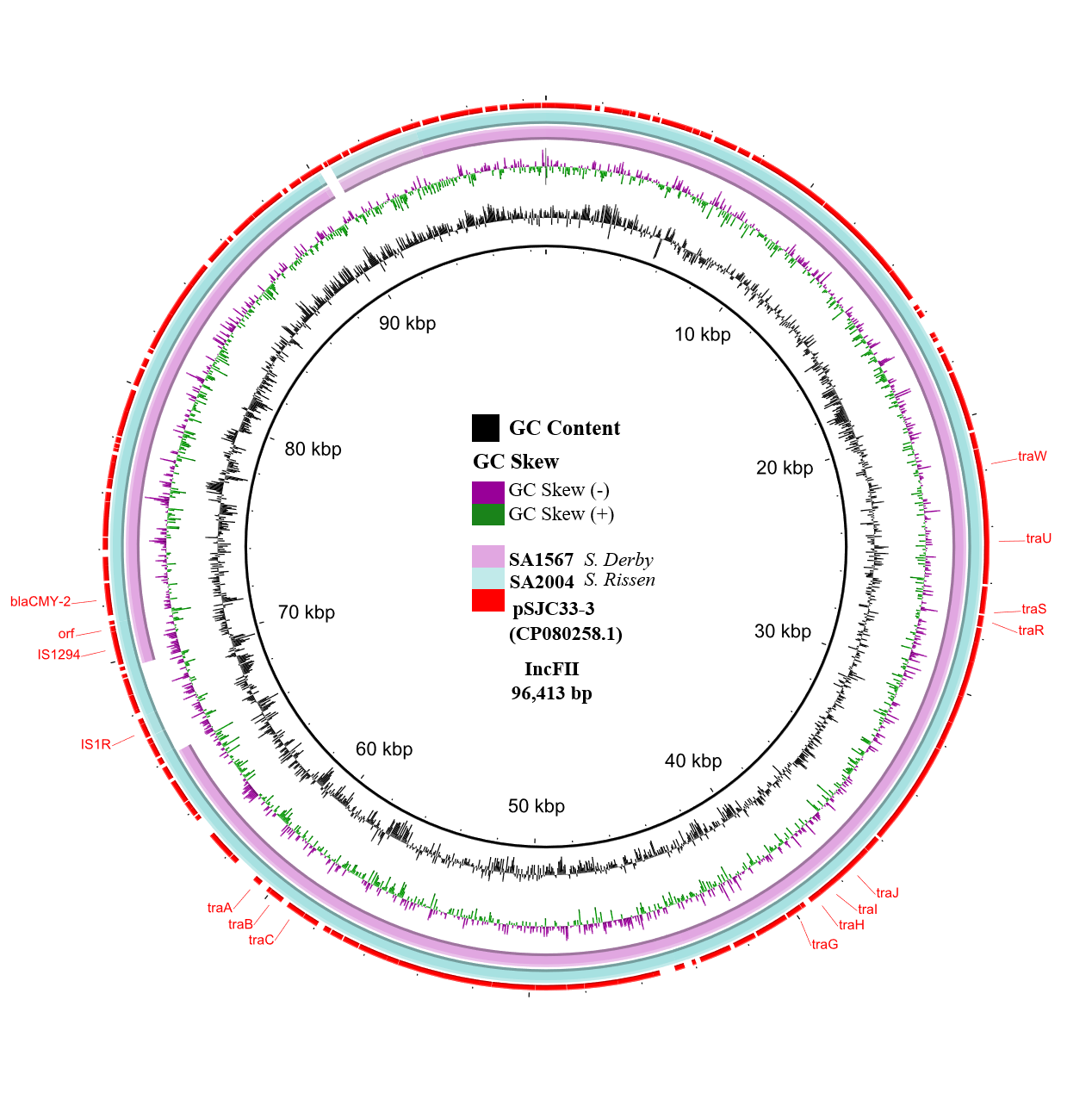


**Supplementary Figure S9. Alignment of a *bla*_CMY-2_-bearing plasmid from foodborne *Salmonella* in this study with a similar IncFII plasmid by BRIG.** Illumina contigs of a CMY-2 bearing plasmid show high similarity to the IncFII plasmid pSJC33-3 (GenBank accession number CP080258.1) in the NCBI database.
